# Paleogeographic controls on Phanerozoic weathering rates and implications for climate regulation

**DOI:** 10.1038/s41467-026-77289-3

**Published:** 2026-09-03

**Authors:** R. J. Graham, B. B. Cael, M. Fu, D. S. Abbot

**Affiliations:** 1 Department of Geophysical Sciences, University of Chicago, Chicago, IL USA; 2 School of Ocean and Earth Science, University of Southampton, Southampton, UK

**Keywords:** Palaeoclimate, Carbon cycle

## Abstract

Earth’s climate has remained habitable for billions of years, a stability usually attributed to a negative feedback from silicate weathering – the chemical breakdown of silicate rocks on land, which consumes atmospheric carbon dioxide faster as climate warms. However, whether temperature or hydrology primarily drives this feedback remains uncertain. Here we show the two controls leave geographic fingerprints: in an ensemble of climate simulations with randomized continents, temperature-controlled weathering depends on tropical land fraction, whereas hydrology-controlled weathering depends on continental fragmentation. Stronger weathering removes more carbon dioxide, cooling the planet, so each regime predicts anti-correlation between temperature and its controlling geography. Reconstructed global temperatures spanning 480 million years are anti-correlated with tropical land fraction, except from roughly 295 to 200 million years ago – overlapping the supercontinent Pangea – when they anti-correlate with continental fragmentation. The mechanism stabilizing Earth’s climate may thus have evolved, with temperature control apparently dominant for most of this period.

## Abstract (web summary)

Simulations with randomized continents show that temperature- and hydrology-controlled weathering results in climates that track different features of continental geometry. The Earth’s record suggests the control may have shifted over 480 million years.

## Introduction

Earth’s climate is regulated on long (10^5^ to 10^6^ year) timescales by stabilizing negative feedbacks within the carbonate-silicate cycle^1,2^. Silicate weathering reactions on Earth’s surface and seafloor release solutes that enter the ocean and react with oceanic carbon that is in chemical equilibrium with CO_2_ in Earth’s atmosphere to form carbonate minerals. This process is the primary inorganic sink of CO_2_ outgassed from Earth’s interior, and a combination of theoretical arguments, laboratory experiments, and observational evidence[e.g., refs. ^1,3–5^] suggest that weathering reactions accelerate under warmer conditions, providing a feedback mechanism to regulate Earth’s climate: When the planet warms, silicate weathering speeds up, accelerating the removal of CO_2_ from the atmosphere. This carbon cycle response leads to CO_2_ drawdown and climatic cooling, which in turn slows the silicate weathering rate until CO_2_ is being removed from the atmosphere at the same pace that CO_2_ is added by volcanic outgassing. The same process happens in reverse in response to climatic cooling. Although transient imbalances between CO_2_ inputs and outputs due to organic carbon interactions^6^ or limitations in silicate mineral supply^7^ may have played important roles in Earth’s climate evolution, weathering-based coupling between atmospheric CO_2_ removal and background climate appears to play a key role in maintaining the quasi-equilibrium in the carbon cycle necessary to avoid frequent excursions to extremely high or low surface temperatures that would have threatened the existence of complex life on Earth during the Phanerozoic^8,9^.

However, despite a general agreement among researchers about the importance of negative feedback within the carbonate-silicate cycle in sustaining Earth’s habitability, the details of the mechanism(s) mediating this stabilization through Earth's history remain highly uncertain. In this study, we examine a fundamental open question regarding the silicate weathering feedback on land [e.g., refs. ^10–16^]: Is its coupling to the climate mediated primarily by temperature in the weathering zone, or does hydrology play a stronger role? Many attempts to model Earth’s silicate weathering feedback have based their estimates of the temperature-dependence of Earth’s weathering rate on direct laboratory measurements of the kinetics of silicate dissolution[e.g., ref. ^17^]. But some recent modeling and theoretical studies have argued that the apparent global temperature-dependence of silicate weathering is significantly weaker than would be expected from laboratory measurements[e.g.^4,5,18^]. One potential explanation for this discrepancy is that the feedback between climate and weathering may not be rooted directly in the kinetics of silicate dissolution, but is instead a result of the climate-dependence of the flux of water through the weathering zone^12,19^. Calculations of weathering rates that account for physicochemical interactions between water, dissolving silicates, and the secondary minerals (clays and silica) that precipitate during silicate weathering suggest that, depending on the local lithology, silicate weathering reactions can reach a chemical equilibrium where solutes in runoff are saturated, preventing further net silicate dissolution until unsaturated water flows through–a regime sometimes referred to as “chemostatic” or “runoff-limited” weathering^12,20–22^. In this regime, climate influences weathering through its influence on hydrologic intensity, with greater fluxes of water leading to proportional increases in silicate weathering, largely independent of local temperature. Since global runoff has a much weaker dependence on temperature than the kinetic temperature dependence of silicate weathering, hydrologically regulated weathering leads to a weaker apparent temperature-dependence and a significantly less effective thermostat compared to silicate weathering based on laboratory measurements of silicate dissolution^19^.

Although the behavior of silicate weathering and its interaction with the climate is still subject to debate, a substantial volume of work attempts to connect changes in paleogeography during the Phanerozoic Eon to changes in climate and CO_2_ through the weathering feedback using models of widely varying complexity [e.g., refs. ^23–32^]. Early models, such as refs. ^23,24^ assumed a strong temperature control on weathering with no direct runoff-dependence and used bulk estimates of land area and average landmass latitude to predict climate as a function of paleogeography. The GEOCARB and GEOCARBSULF lineage of global-mean box models^26^ used relationships derived from GCM studies like that of^33^ to represent the impact of paleogeography on global-mean runoff and temperature, allowing for an effect on climate through a global weathering relationship based on both an assumed strong temperature sensitivity and a moderate runoff sensitivity. The GEOCLIM lineage of models[e.g., refs. ^29,30^] and successors like SCION^31^ couple weathering parameterizations with strong temperature-dependence and strong (linear) runoff-dependence to output from GCM simulations in order to calculate spatially resolved weathering fluxes, allowing for investigations of climate and CO_2_ evolution that directly account for paleogeographic changes in Earth history[e.g., refs. ^27,28,34–39^]. However, these models all take their chosen weathering feedback strengths for temperature and runoff as given (modulo occasional sensitivity tests), introducing a major source of uncertainty into their results and reconstructions.

Here, to assist in differentiating between signatures of hydrology-controlled and temperature-controlled weathering in Earth history, we analyze a large ensemble of GCM simulations that are identical except for their randomized continental configurations and post-process their runoff and temperature fields to estimate global weathering rates from them. This allows us to demonstrate that these alternative weathering controls lead to alternative predictions for Earth’s climate response to changes in aspects of bulk planetary continental geometry. Then, we statistically compare proxy-based reconstructions of Earth’s climate and land configuration to assess whether the geologic record supports one hypothesis over the other. Ultimately, our calculations are consistent with the hypothesis that Earth’s weathering control mechanism shifted twice in the last 480 Ma, from a temperature-based to a hydrology-based configuration  ≈ 300 Ma and then back to a temperature-based configuration  ≈ 200 Ma, though this relies on assumptions like weakly varying outgassing, a tight relationship between CO_2_ and surface temperature, and the accuracy of uncertain paleogeographic reconstructions. Still, the results provide useful new insight into the structure of the carbon cycle which may lead to improved understanding of the evolution of Earth’s climate over the Phanerozoic.

## Results

### Sensitivity of silicate weathering to tropical land fraction and continental fragmentation

**Fig. 1:**
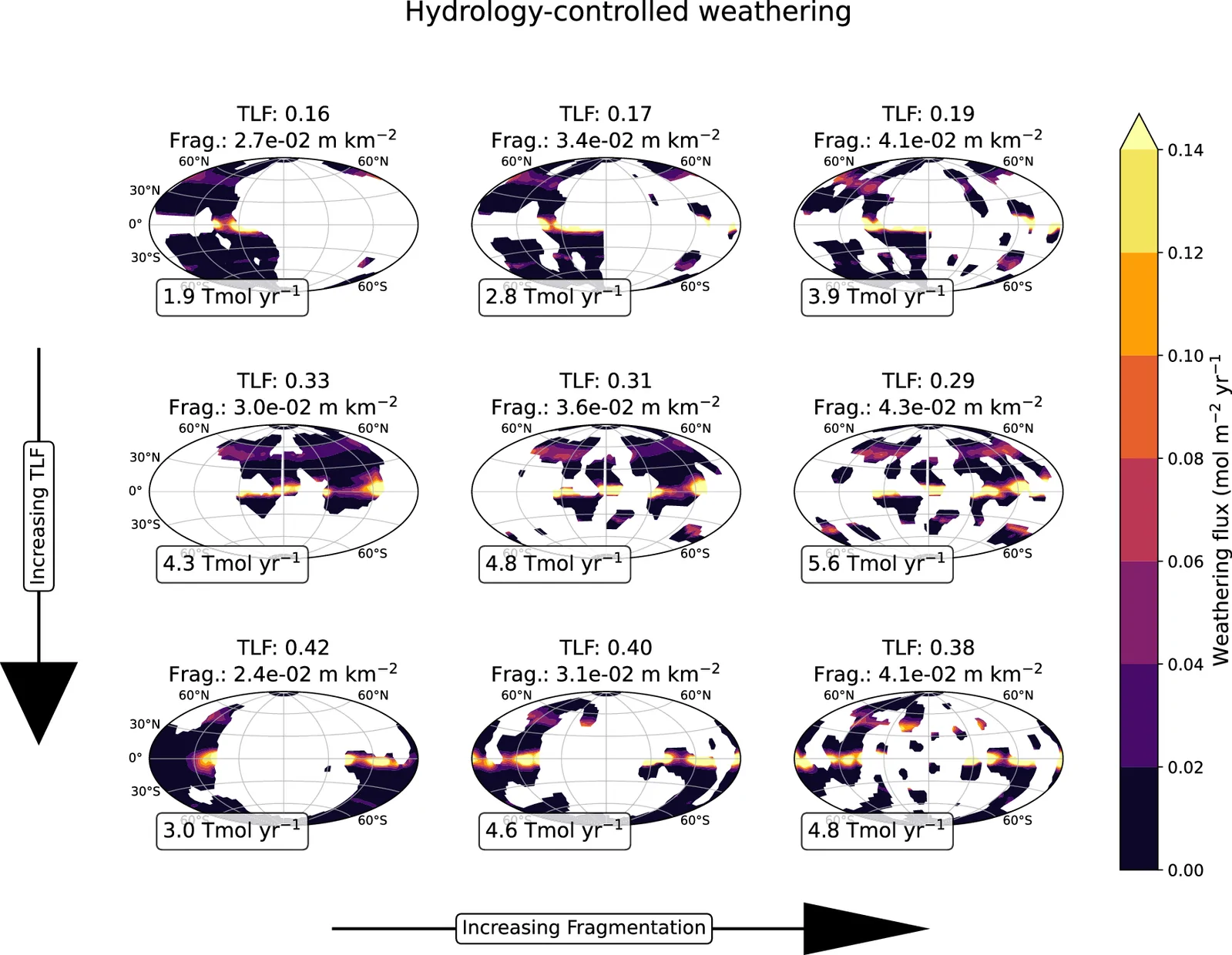
Example continental configurations and hydrology-controlled weathering outputs from the global climate model (GCM) ensemble. White areas denote the ocean, and colored areas denote land, with coloration mapping onto local weathering flux. Each map is labeled with its total global weathering rate in Tmol yr ^−1^ in a white box in its bottom left corner. Left column: Continental configuration with small fragmentation index (supercontinental). Middle column: Configuration with medium fragmentation index (partially fragmented). Right column: Configuration with large fragmentation index (dispersed). Each row of three configurations shares one of the 40 initial random sets of spherical harmonic coefficients (see Methods for more information), allowing them to represent a sequence of snapshots from the assembly of the supercontinent in the right panel. The top row has the smallest tropical land fraction (TLF), the middle row has middling TLF, and the bottom row has the largest TLF. Weathering fluxes in each grid cell were calculated with *α* = 1 (linear correspondence between runoff and weathering) and *k* = 0 K^−1^ (no ditemperature dependencedence for weathering) in eqn. (2).

**Fig. 2:**
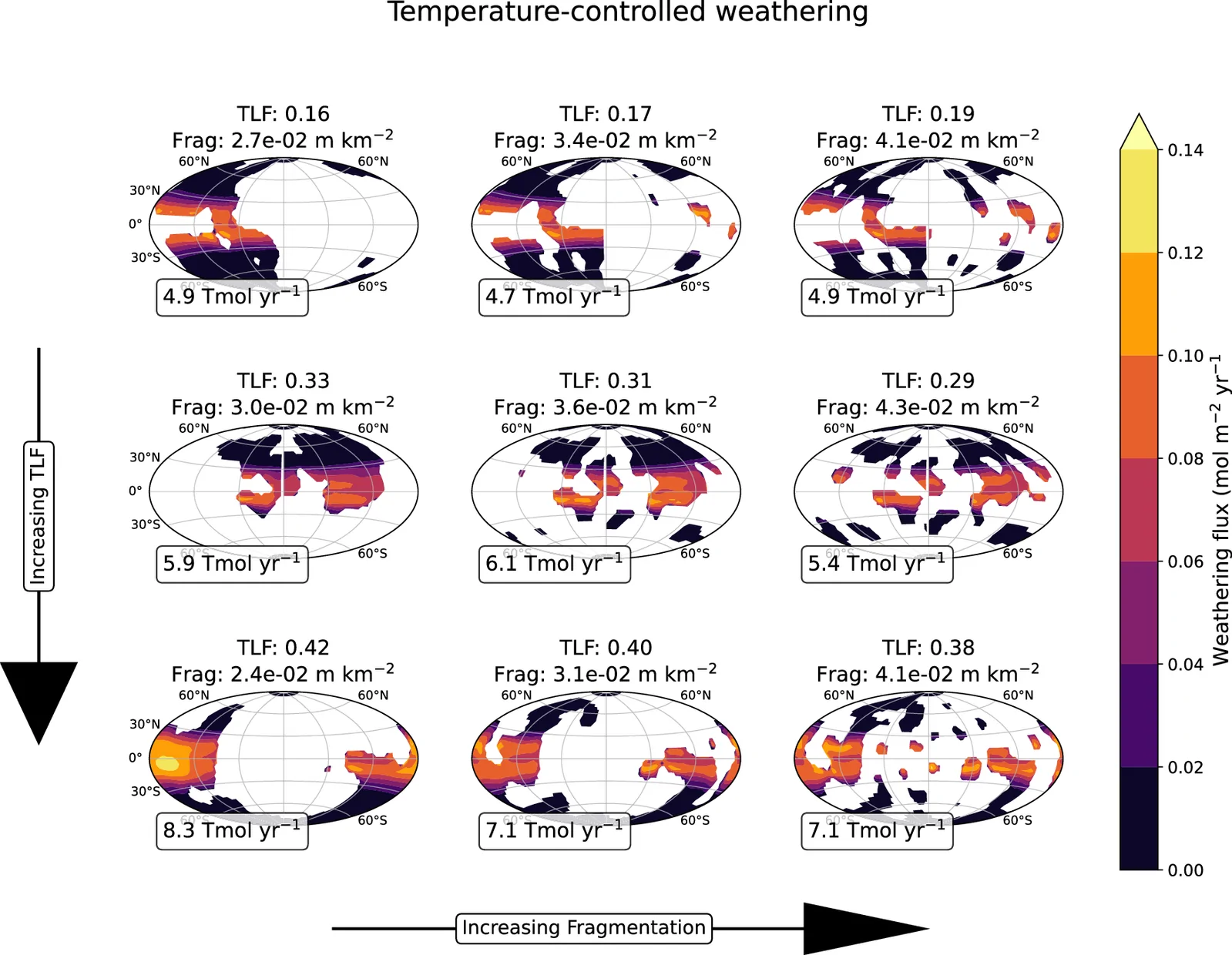
Example continental configurations and temperature-controlled weathering outputs from the global climate model (GCM) ensemble. White areas denote the ocean, and colored areas denote land, with coloration mapping onto local weathering flux. Left column: Continental configuration with small fragmentation index (supercontinental). Middle column: Configuration with medium fragmentation index (partially fragmented). Right column: Configuration with large fragmentation index (dispersed). Each row of three configurations shares one of the 40 initial random sets of spherical harmonic coefficients (see Methods for more information), allowing them to represent a sequence of snapshots from the assembly of the supercontinent in the right panel. The top row has the smallest tropical land fraction (TLF), the middle row has middling TLF, and the bottom row has the largest TLF. Weathering fluxes in each grid cell were calculated with *α* = 0 (no direct runoff-dependence of weathering) and $k=\frac{1}{11.1}$ (strong direct exponential temperature-dependence of weathering) K^−1^ in eqn. (2).

**Fig. 3:**
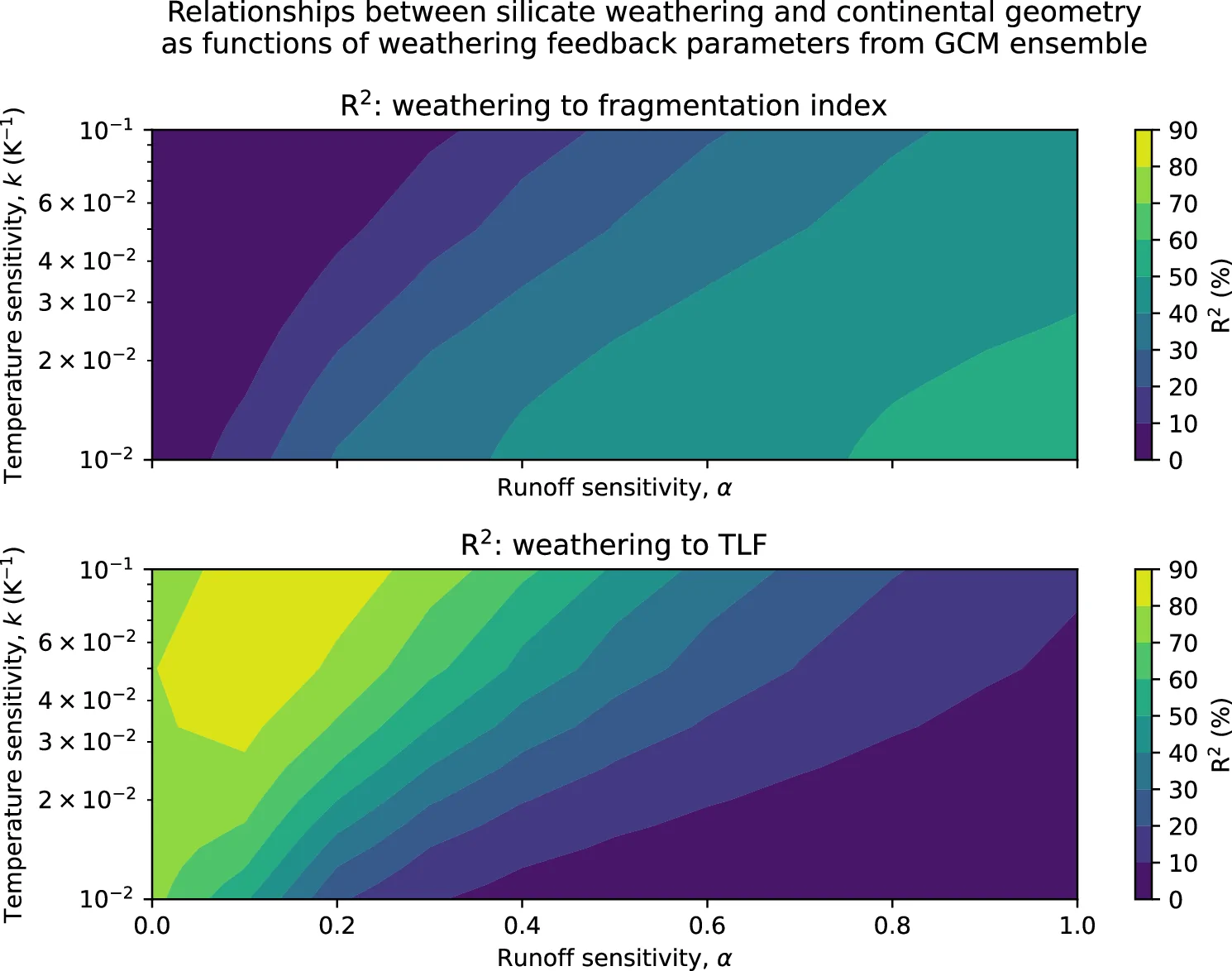
Coefficient of determination between silicate weathering and fragmentation index (top panel) and tropical land fraction (TLF; bottom panel). In each panel, the x-axis represents runoff sensitivity (*α*) and the y-axis represents temperature sensitivity (*k*). Contour color indicates coefficient of determination (R^2^) between weathering and the respective continental geometry variables.

In this section, we present an analysis of the correlations between continental geometry and global weathering rates in an ensemble of idealized GCM simulations that suggests a relationship between planetary processes that might be observable in the geologic record. The key point is that the carbon cycle responds very differently to variations in TLF (defined as the land fraction between 15^∘^N/S for consistency with^40^, with results insensitive to variations in latitude range) and continental fragmentation (defined as continental perimeter divided by continental area) depending on whether the weathering feedback is mediated primarily by hydrology (see Fig. 1), temperature (see Fig. 2), or both (see Fig. 3). Figures 1, 2 both display weathering rates for the same nine land configurations, with the two figures differing only in that weathering varies linearly with hydrology with no direct temperature dependence in Fig. 1, while it varies exponentially with temperature and lacks hydrology dependence in Fig. 2. In both figures, each row of plots displays increasing continental fragmentation moving left to right, and TLF increasing moving downward. From Fig. 1, it is clear that hydrologically controlled weathering increases with increasing continental fragmentation (moving left to right) and is not strongly affected by TLF. On the other hand, temperature-controlled weathering (see Fig. 2) strongly increases with increasing TLF, while the effect of continental fragmentation is minimal or, in the case of the largest TLF cases, may even lead to a weathering increase with decreasing fragmentation (i.e., bottom row, Fig.2).

We note for clarity that these weathering values are all calculated with the same background CO_2_ and global temperature and might therefore be better thought of as values of bulk “global weatherability,” since Earth’s climate would in reality adjust to bring the global weathering rate into balance with the background CO_2_ outgassing rate if a change to continental geometry were to cause a large transient shift in weathering. In other words, these calculations are not intended to imply that the equilibrium global weathering rate has varied by this magnitude over Earth history (since the equilibrium global rate of weathering is set by outgassing), but instead to illustrate the degree to which changes in continental geometry can act as carbon cycle forcings under different assumptions about global weathering controls^41^. Several studies have identified changing “weatherability” as a potential major driver behind climate changes during the Phanerozoic Eon due to its influence on the equilibrium point of the carbon cycle and climate system^42–46^.

To quantify the apparent relationships between weathering and continental geometry, we calculated coefficients of determination between the global weathering rate and TLF and continental fragmentation for our GCM ensemble over a wide range of values for *k* (temperature-dependence of weathering; see eqn. (2) in Methods) and *α* (runoff-dependence of weathering; see eqn. (2)), with results shown in Fig. 3. When the weathering feedback is dominated by hydrology instead of temperature (large *α*, small *k*), the coefficient of determination between the total global weathering rate (*W*_tot_) and the fragmentation index, R^2^(*W*_tot_, fragmentation index), can exceed 50% (bottom right corner of top panel in Fig. 3), while R^2^(*W*_tot_, TLF) falls to nearly zero (bottom right corner of bottom panel in Fig. 3). Conversely, when temperature regulation dominates over hydrology (small *α*, large *k*), R^2^(*W*_tot_, fragmentation index) falls to near zero, while R^2^(*W*_tot_, TLF) climbs to more than 75%.

**Fig. 4:**
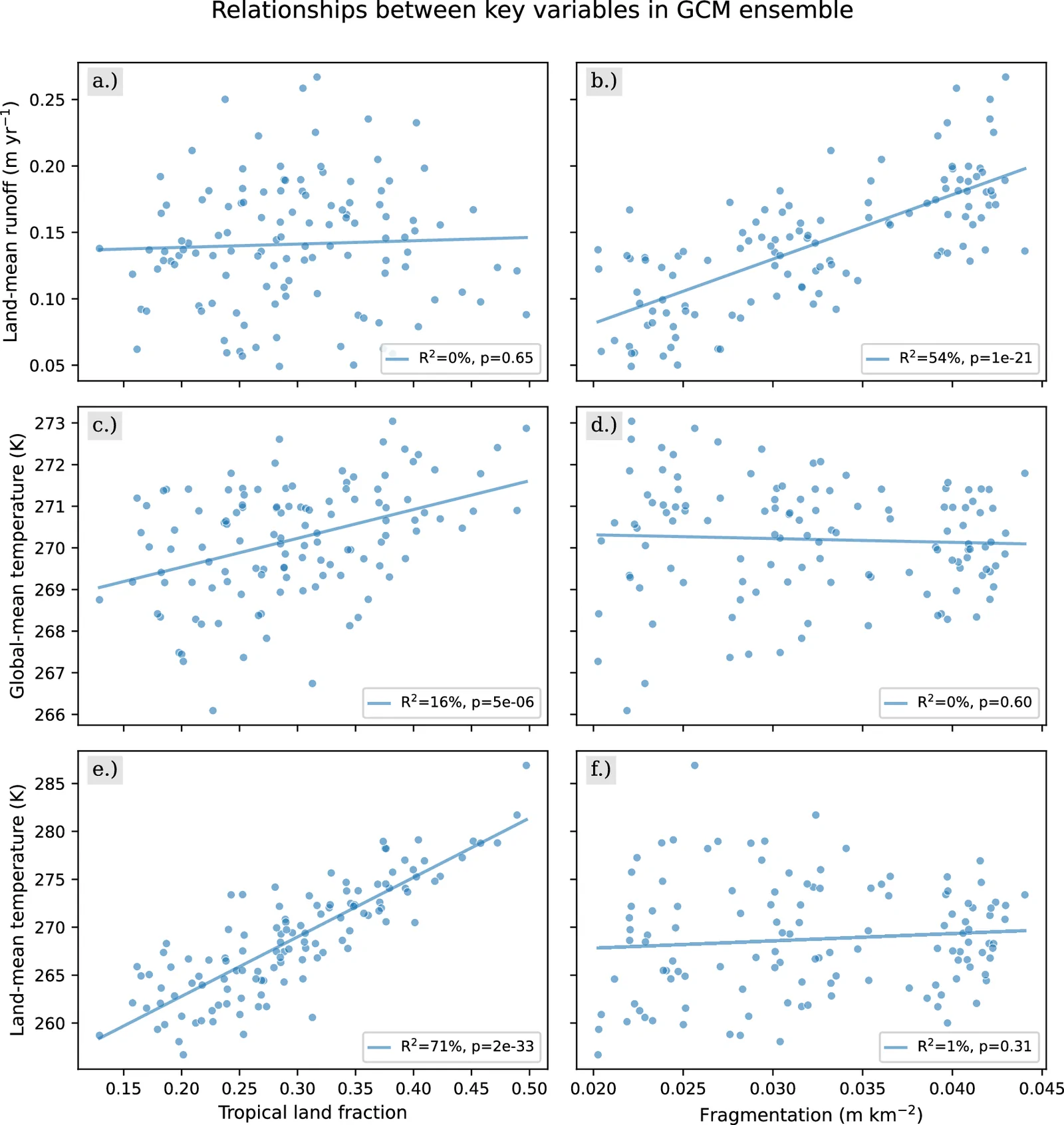
Scatter plots of average values of different variables from the global climate model (GCM) ensemble. **a** shows the lack of correlation between runoff and tropical land fraction (TLF); **b** shows a relatively strong correlation between runoff and fragmentation; **c** shows a weak correlation between global-mean surface temperature and TLF; **d** shows no correlation between global-mean surface temperature and fragmentation; **e** shows a strong correlation between global-mean land temperature and TLF; and **f** shows a lack of correlation between global-mean land temperature and fragmentation.

The reason for this divergence in response to continental geometry is straightforward. Within the GCM ensemble, global runoff is uncorrelated with TLF (R^2^ = 0%) but strongly correlated with continental fragmentation (R^2^ = 54%; see Fig. 4a, b). The lack of correlation between TLF and runoff is due to an increase in land evaporation in high-TLF simulations that compensates for increased land precipitation, similar to an effect observed by ref. ^33^. This occurs because tropical land intercepts more solar energy, allowing for more efficient recycling of water despite increased overall delivery to the land via precipitation in the wet tropics. Increasing fragmentation does not increase the ease of delivery of solar energy to land, but it does increase delivery of water, so runoff tends to increase as continents break up. So, when weathering is dominated by hydrology, changes to TLF have a minimal effect due to small changes in runoff, but more fragmented configurations produce more runoff, accelerating weathering.

Conversely, although global-mean surface temperature is weakly correlated with TLF (R^2^ = 16%) and uncorrelated with continental fragmentation (R^2^ = 0%; see Fig. 4c, d), global-mean land temperature is strongly correlated with TLF (R^2^ = 71%), while remaining uncorrelated with fragmentation (R^2^ = 1%; see Fig. 4e, f). So, when temperature is the main driver of weathering, changes to TLF can change the weathering rate by altering the amount of land that experiences higher temperatures near the equator. The small positive relationship between global-mean surface temperature and TLF visible in Fig. 4c is due to an increase in high-albedo land snow in low-TLF simulations where land is colder and closer to the poles, since snow on land has higher albedo than sea ice in these simulations. Warmer global conditions moderate this effect: a smaller ensemble of simulations with 30 of the 120 paleogeographies from this paper and^40^ with doubled CO_2_ (2000 ppm) showed a TLF-global temperature correlation with an even weaker R^2^ of 2% (see Supplementary Fig. 4 in Supplementary Information Section A.4) due to the reduced latitudinal extent of freezing temperatures while preserving a strong TLF-land temperature correlation. In general, the 30 warmer simulations showed the same basic behavior and relationships as the simulations seen in Fig. 4, as did a set of 30 colder simulations (500 ppm CO_2_) with the same subset of paleogeographies as the warmer simulations (see Supplementary Fig. 4 in Supplementary Information Section A.4).

These distinctive differences in the response of the weathering feedback to continental geometry naturally lead to a hypothesis: If Earth’s carbonate-silicate thermostat is dominated by surface weathering that is primarily sensitive to surface temperatures, then, for a given global outgassing rate, we should observe an anticorrelation between TLF and surface temperature through time, because an increase in TLF would be expected to increase the weathering rate and cause CO_2_ drawdown and cooling. Similarly, a reduction in TLF would slow weathering and allow CO_2_ to accumulate, leading to warming. Furthermore, we should not expect there to be a strong correlation between climate and continental fragmentation in this regime. Alternatively, if the silicate weathering response is dominated by Earth’s hydrological response to changes in climate, for a given outgassing rate and with all else being equal, we should expect an anticorrelation between continental fragmentation and climate and little influence from TLF. These opposing predictions provide a geological test that might shed light on the mechanisms that have stabilized Earth’s climate throughout planetary history. In the following section, we investigate Earth’s geologic record in the context of these findings.

### Correlations between surface temperature and continental geometry over the Phanerozoic

**Fig. 5:**
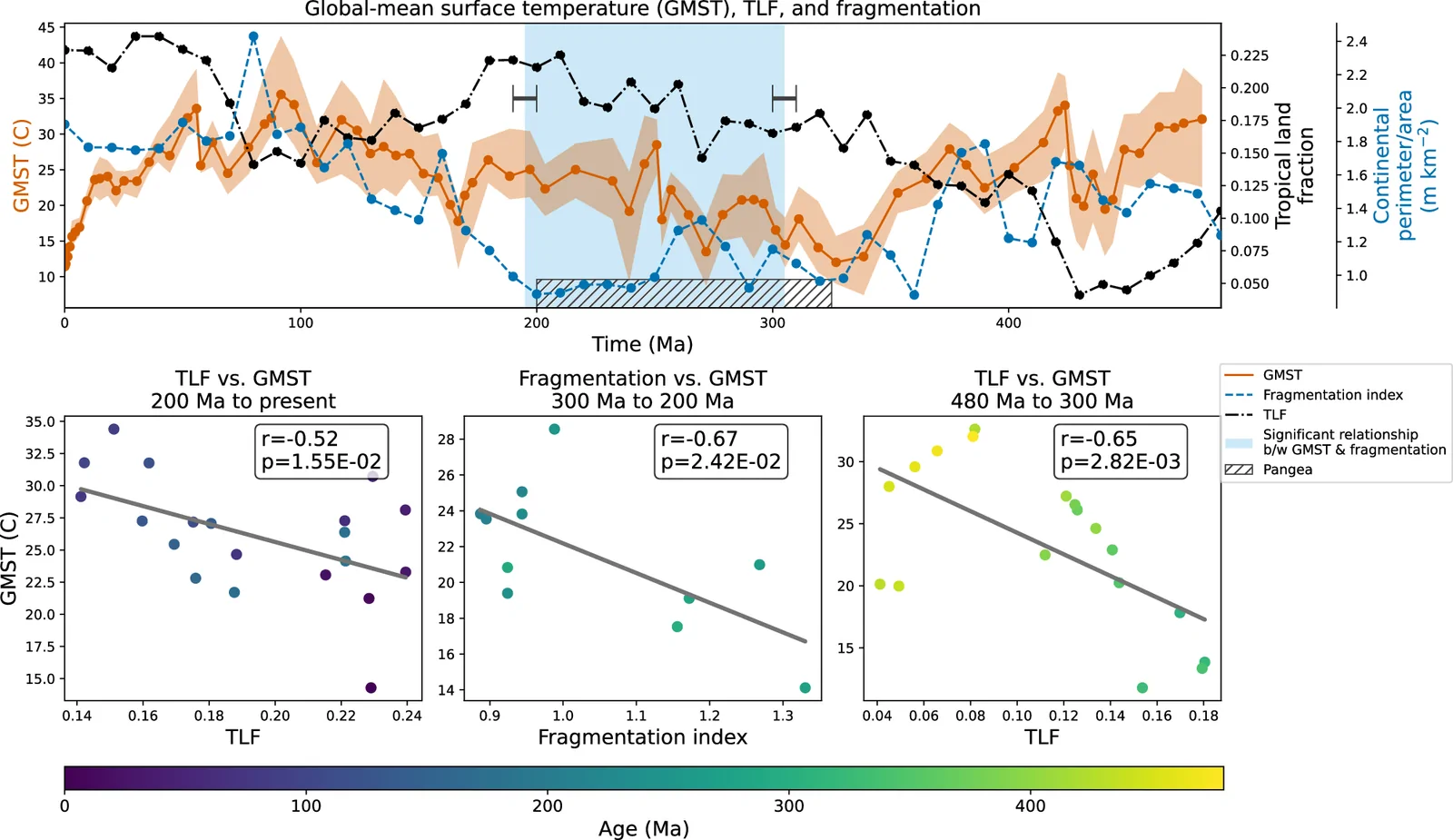
Time series of reconstructed global-mean surface temperature (GMST), tropical land fraction (TLF), and continental fragmentation (top panel) and scatter plots of GMST vs. TLF and fragmentation index (bottom panels). In the top panel, GMST^47^ is the red curve, TLF^48^ is the black dash-dotted curve, and continental fragmentation is the dark blue dashed curve. The translucent red envelope represents the 95% confidence interval for GMST from ref. ^47^. The light blue region in the top panel represents the period of continental fragmentation and GMST are significantly anti-correlated and the white regions are where TLF and GMST are significantly anti-correlated, with the error bars covering the range of times where the data allow for switches between the two modes (based on significant relationships between GMST and TLF or fragmentation, see Supplementary Information Section A.1). The hatched region covers the period during which Pangea existed^49^. The bottom three panels show correlation between GMST and (from left to right) TLF from 200 Ma to present, fragmentation index from 300 Ma to 200 Ma, and TLF again from 480 Ma to 300 Ma, with gray lines marking the lines of best fit, Pearson correlation coefficients in the upper right, and markers colored according to age.

To differentiate between the hypotheses described in the previous section, we analyzed the statistical relationships between the GMST reconstruction of^47^ and continental fragmentation and TLF calculated from the paleogeographic reconstruction of^48^ (see Fig. 5). We find that from 480 Ma to 295  ± 15 Ma (the range of possible transition times is shown as the rightmost error bar in Fig. 5), GMST displays a strong (*r* < − 0.5) and significant (*p* < 0.025) anti-correlation with TLF and no significant relationship to fragmentation. Then, at 295  ± 15 Ma, the apparent correlation switches, and subsequently GMST displays a strong, significant anti-correlation with continental fragmentation and no significant relationship to TLF (blue shaded region in Fig. 5) until 200  ± 10 Ma (leftmost error bar range in Fig. 5). Finally, from 200  ± 10 until the present, the relationships between variables revert back to their original behavior, with anti-correlation between GMST and TLF and no significant relationship between GMST and fragmentation. The period of anti-correlation with continental fragmentation notably overlaps with Pangea, which lasted from  ≈ 325 Ma to  ≈ 200 Ma[colored pink in Fig. 5,^49^]. The negative relationships between GMST and TLF and the fragmentation index during the three distinct periods are visible in the bottom three panels of Fig. 5.

The evolution of the relationships between GMST, TLF, and continental fragmentation is consistent with a dynamic scenario in which the main mechanism controlling the coupling between silicate weathering and climate has shifted across the Phanerozoic. Based on our GCM ensemble results, the anti-correlation between GMST and TLF at the beginning and end of the period under examination suggests temperature-controlled weathering, while the anti-correlation between GMST and fragmentation from  ≈ 300 Ma to  ≈ 200 Ma suggests that hydrology dominated the weathering response during that time span.

### Discussion

As a sensitivity test of our results, we repeated the analysis of correlations between surface temperature and exposed continental geometry over the Phanerozoic, replacing the paleogeographic reconstructions from ref. ^48^ with those of ref. ^50^. The two sets of reconstructions differ primarily due to different underlying plate tectonic models^48^, although they are not entirely independent, since ref. ^48^ relied on some of the same data sources as ref. ^50^. With the Scotese reconstruction (see Supplementary Fig. 2), our results are qualitatively similar, with a significant negative relationship between TLF and GMST from 480 to 300 Ma (*r* = − 0.70, *p* = 2.7 × 10^−6^) and a significant negative relationship between fragmentation and GMST from 300 Ma to 200 Ma (*r* = − 0.62, *p* = 2.7 × 10^−3^), but the anti-correlation between TLF and GMST from 200 Ma to present is weaker (*r* = − 0.31) and marginally significant (*p* = 0.054).

Analyzing reconstructed CO_2_ may seem like a more direct route for demonstrating a relationship between climate and paleogeography, since changes to silicate weathering fluxes should manifest as changes to CO_2_ levels. While there do exist a number of CO_2_ records over the Phanerozoic based on modeling and data compilations^51–53^, pre-Cenozoic CO_2_ estimates are poorly constrained and often inconsistent^54^. Given the large uncertainties associated with CO_2_ paleo-records and the fact that Phanerozoic temperature variations are thought to mainly reflect CO_2_ forcing^55,56^, we choose to focus on GMST in this study. In any case, the reconstructed GMST of^47^ correlates strongly with (log-transformed) reconstructed CO_2_ over most of the Phanerozoic [*r* = 0.77, *p* < 0.01,^47^], and our GCM ensemble displayed weak or non-existent relationships between global-mean surface temperature and both TLF and continental fragmentation (Fig. 4c, d). This increases our confidence that it is a reasonable first-order assumption that CO_2_ and temperature were tightly linked over most of the Phanerozoic and that variations in continental geometry were unlikely to have a large direct (non-carbon-cycle-mediated) effect on surface temperatures, consistent with previous GCM ensemble studies [e.g., refs. ^33,57^].

It is also important to note that our GCM simulations and weathering calculations are idealized in ways that could impact the results of this study. For example, the land topography is muted compared to Earth, with a maximum elevation of 100 m^40^. This means there are no mountain ranges, so our results do not include the potentially important impact of orographic precipitation^58^ or possible interactions between uplift, erosion, and weathering^59–62^, which may vary systematically with continental fragmentation. We also did not attempt to explicitly represent variations in lithology, which could produce large variations in weathering parameters across the planet at any given time [e.g., refs. ^22,63,64^]. Additionally, the weathering calculations do not account for the potential biotic acceleration of silicate weathering by land plants [e.g., refs. ^65–67^], though it is unclear what impact the rise of forested ecosystems  ≈ 385 Ma had on chemical weathering^68,69^. Furthermore, glacial weathering may be an important component of CO_2_ drawdown^70^, so since ice extent has varied during the past 485 Ma, glacial cover could provide another co-varying modulator of weathering rates that could impart spurious correlations to our data, with glacial periods during the Ordovician-Silurian, end-Carboniferous, and late Cenozoic^71^. Seafloor weathering is also absent from our model, despite indications that it may play a crucial role in Earth’s climate stabilization similar to that of silicate weathering on the land^2,72,73^. Similarly, we do not consider authigenic clay formation ("reverse weathering”) which consumes alkalinity and could impact climate equilibria and the response of the carbon cycle to perturbations [e.g., refs. ^74–77^].

More generally, given the interconnected and recursive nature of the Earth system, it is difficult to establish causality when analyzing relationships between geological quantities. Although we observe correlations between the variables of interest in this study (i.e., GMST, TLF, and continental fragmentation), it is possible that these relationships are being driven by other mechanisms or variables that are co-varying simultaneously. For example, we excluded variations in CO_2_ outgassing rate from our study even though such variations could have played a role in dictating the equilibrium weathering rate and climate state at any given time in Earth history. This is justified given the major uncertainty in reconstructions, with large inconsistencies in the magnitude and direction of outgassing changes depending on the set of processes considered and the methodology used for estimation^30,31,48,71,78–82^. Still, theoretical arguments and some data suggest outgassing may vary systematically across the supercontinent cycle, with periods of enhanced rifting corresponding to increases in the flux of CO_2_ from Earth’s interior [e.g., refs. ^83–85^]. If CO_2_ outgassing increases when continental fragmentation increases, then this may systematically bias the system away from displaying an anti-correlation between the degree of fragmentation and global surface temperature, since increased weathering could be partially or completely offset by increased outgassing. If that is the case, then the imprint of hydrologically controlled weathering on the geologic record might be reduced, biasing our analysis.

One potential geologic test of the hypothesis that the climate stabilization mechanism has shifted from temperature-dependence to hydrology-dependence and back during the Phanerozoic would be to examine global spatial and temporal trends in correlations between paleosol-based precipitation and temperature proxies with weathering proxies like chemical index of alteration^86,87^ or the weathering index of Parker^88^. Deng et al.^15^ used this method (except with direct temperature and precipitation measurements rather than proxies) to infer a strong temperature control on silicate weathering and a weak hydrologic control for the modern Earth, consistent with the results of our study. It is an open question whether geologic proxies would reveal the same pattern on Earth throughout the Phanerozoic across the period our analysis suggests may have displayed hydrologically-dominated weathering. Paleosols^89^ and GCM simulations^29,90^ of Earth during Pangea [which existed from  ~ 325 Ma to  ≈ 200 Ma,^49^] suggest that this would have been a period of unusually low global precipitation and runoff, which would tend to push weathering zones toward hydrologic control by reducing water fluxes and allowing solutes to build to higher concentrations^12,64^. This observation suggests that the aridity of the Pangea supercontinent might explain the potential shift to hydrologic control described in this study.

Beyond Earth’s history, if Earth’s thermostat oscillates between temperature control and hydrology control over time due to the supercontinent cycle (or because of some other driver), then in addition to being crucial for understanding Earth’s past, this effect could be important for accurately predicting the future lifespans of various components of Earth’s biosphere^91,92^. This is because (in the absence of unpredictable biotechnological intervention) Earth’s far future climate state will be determined by the interplay between the gradually brightening Sun and the behavior of atmospheric CO_2_ in response to this forcing, as dictated by the carbonate-silicate cycle, so the details of silicate weathering processes will be a primary control^91–93^. Similarly, the classical “circumstellar habitable zone” is typically defined as the band of orbits around a star where rocky exoplanets with CO_2_-N_2_-H_2_O atmospheres can in principle sustain liquid water oceans with the appropriate quantity of CO_2_ in the atmosphere^94,95^, and this concept is rooted in the idea that sufficiently Earth-like planets will have carbonate-silicate cycles capable of regulating their CO_2_ within bounds that allow for liquid water like Earth^96^. Some even propose to limit the width of the habitable zone based on simulations that suggest that silicate weathering behavior can cause periodic global glaciations for planets receiving somewhat less sunlight than Earth, but the precise details of the weathering parameterization used strongly impact the resulting climate behavior [e.g., refs. ^97–99^]. Therefore, the question of whether temperature or hydrology controls weathering on Earth directly affects predictions for the climates of potentially habitable exoplanets^19,22,100,101^.

In this study, we used weathering trends inferred from post-processing a large ensemble of GCM simulations to propose a pair of relationships between components of Earth’s continental geometry and silicate weathering behavior: If silicate weathering is primarily controlled by the local temperature in the weathering zone, then weathering should correlate strongly with tropical land fraction, but if weathering is controlled by hydrology, then it should correlate strongly with continental fragmentation. In turn, those relationships imply that temperature-controlled weathering should result in an anti-correlation between global-mean surface temperature and tropical land fraction, while hydrology-controlled weathering should result in an anti-correlation between global-mean surface temperature and fragmentation. We then analyzed Earth’s geologic record over the past 480 Myr and found that these relationships between continental geometry and climate seem to manifest in distinct periods: From 480 to 295 ± 15 Ma, surface temperature and tropical land fraction are anti-correlated, then from 295 ± 15 to 200 ± 10 Ma surface temperature and continental fragmentation are anti-correlated, and finally from 200 ± 10 Ma to the present surface temperature and tropical land fraction appear anti-correlated once again. The apparent shift in the mechanism stabilizing Earth’s climate overlaps with the existence of Pangea, suggesting a potential relationship between Earth’s dominant weathering mode and the mode of Earth’s supercontinent cycle, perhaps mediated by large changes in continental aridity.

## Methods

### Climate simulations

We use an ensemble of Community Earth System Model (CESM) global climate model (GCM) simulations originally presented by ref. ^40^ and available for download at ref. ^102^ to analyze the sensitivity of silicate weathering rates to tropical land fraction (TLF) and continental fragmentation, represented by the ratio of non-submerged continental perimeter to continental area, which we denote the “continental fragmentation index” following^103^. The GCM simulations were all carried out at T31 resolution ( ≈ 4^∘^ by 4^∘^), and all have an identical top-of-atmosphere subsolar insolation of 1285 W m^−2^, global land fraction of 30%, atmospheric CO_2_ concentration of 1000 ppm (parts per million by volume), obliquity of 23.5^∘^, eccentricity of 0, and lack of vegetation. They differ only in their continental geometries, which are stochastically generated spherical harmonics truncated at degree 12. For each land configuration^40^, generated a random set of spherical harmonic coefficients, then weighted them according to:

1 $$S\propto {d}^{p}$$

where *S* is the sum of squared coefficients in each expansion degree *d* and *p* represents the signal processing “color” of the expansion, where for example *p* = − 1 would correspond to “pink noise”. As *p* values become more negative, higher frequency terms are increasingly suppressed. The simulations from^40^ use continental boundary conditions with *p* = − 3 to approximate consolidated supercontinents, *p* = − 2 to approximate partly fragmented continents in the process of coming together or breaking apart, and *p* = − 1 to approximate fully fragmented continental configurations. There are 40 initial random coefficient sets, resulting in a total of 120 continental configurations after each initial set is duplicated and adjusted to create three harmonics with *p* = − 1, *p* = − 2, and *p* = − 3 according to Eqn. (1). Therefore, there are 40 sets of three configurations each that differ in the slope of their power spectrum but not in the relative values within each degree, preserving the qualitative shape of the continents and approximating snapshots of a supercontinental breakup or assembly, as in Figs. 1, 2. See the Extended Data from ref. ^40^ for more details on the generation of the GCM continental configurations.

### Silicate weathering

We take silicate weathering to scale as an exponential function of surface temperature (*T*) and a power-law function of runoff (*q*), excluding a parameterization for CO_2_ dependence because all simulations are performed at the same atmospheric CO_2_:

2 $$w	 \propto {\left(\frac{q}{{q}_{{{\rm{ref}}}}}\right)}^{\alpha }\exp (kT) \\ 	={w}_{{{\rm{ref}}}}{\left(\frac{q}{{q}_{{{\rm{ref}}}}}\right)}^{\alpha }\exp \left(k(T-{T}_{{{\rm{ref}}}})\right)$$

where *w* [mol m^−2^ yr^−1^] is the local silicate weathering flux; *q* [m yr^−1^] is the local runoff output by the GCM; *α* is the runoff sensitivity of silicate weathering; *T* [K] is the local surface temperature output by the GCM; *k* [K^−1^] is the temperature sensitivity of weathering; *w*_ref_ = 0.05 [mol m^−2^ yr^−1^] is “weatherability”[e.g., ref. ^42^] tuned to a value that leads the GCM simulations to produce global weathering rates mostly within the range of  ≈ 3–10 Tmol yr^−1^, based in estimates of global CO_2_ outgassing and analysis of river chemistry [e.g., refs. ^79,104–106^]; *q*_ref_ = 0.26 [m yr^−1^] is a reference global-mean runoff attained by dividing the estimate for Earth’s total modern runoff from ref. ^107^ (38,452 km^3^ yr^−1^) by Earth’s land area ( ≈ 148 × 10^6^ km^2^); and *T*_ref_ = 288 K is a pre-industrial reference temperature^40^. This is a variant of the classic “WHAK”^1^ formulation of continental silicate weathering, a widely used empirical expression to quantify CO_2_ drawdown as a function of climate variables in geologic carbon cycle models[e.g., ref. ^108^]. Models use a wide range of values for the temperature–and runoff-sensitivity parameters under investigation in this study, based on estimates from lab, field, geologic proxies, and theory^1,4,26,53,109,110^.

The total global weathering rate for a given simulation is calculated by summing the local weathering flux multiplied by the land area in each grid cell:

3 $${W}_{{{\rm{tot}}}}={\sum}_{i}{A}_{i}{\gamma }_{i}{w}_{i}$$

where *i* is an index for surface grid cells, *A*_*i*_ is surface area of the *i*th grid cell, *γ*_*i*_ is the land fraction in the *i*th grid cell, and *w*_*i*_ is the weathering flux in the *i*th grid cell calculated according to eqn. (2).

Baum et al. ^40^ calculated weathering rates with a fixed set of sensitivity parameters (*α* = 1 and *k* = 0.09 K^−1^), which limits the applicability of their results due to the large uncertainties in temperature- and hydrology-dependence for silicate weathering. We extend their work by varying *α* across the range *α* ∈ [0, 1] with *α* = 0 corresponding to no runoff-dependence for silicate weathering (except if *q* = 0, we assume weathering shuts off, even if *α* = 0) and *α* = 1 corresponding to a linear dependence. We vary *k* across the range *k* ∈ [10^−2^, 10^−1^] K^−1^, corresponding to a range of *e*-folding temperatures *T*_e_ ∈ [100, 10] K, which encompasses the wide range of weathering temperature sensitivity values that have recently been suggested in the literature^4,5,15^. This wide range of weathering parameters allows us to probe how variations in strength for different forms of coupling between climate and weathering interact with TLF and fragmentation index, two large-scale aspects of continental geometry that may impact global weathering rates. TLF is defined as the land fraction between 15^∘^N/S for consistency with^40^, with results insensitive to variations in latitude range. The fragmentation index is defined as the continental perimeter divided by the continental area. To quantify these relationships, we calculate the coefficient of determination (R^2^) between weathering rates and both TLF and fragmentation across the GCM simulations in our ensemble as functions of *α* and *k* to measure the proportion of variance in weathering rate that is explained by variations in TLF and fragmentation index.

The weathering parameterization we use in this study is based on the common formulation first suggested in ref. ^1^, sometimes referred to as the WHAK model[e.g., refs. ^19,40,111^]. An alternative or extension to the WHAK model presented by ref. ^12^ (MAC) allows for mechanistic transitions between the weathering regimes explored here, with a temperature-controlled regime dominated by silicate dissolution kinetics and a hydrologically regulated regime that occurs when secondary mineral precipitation in the weathering zone prevents solutes from accumulating to higher concentrations. Calculations with the MAC model display very similar patterns to results calculated with WHAK: parameter values with temperature-dependent weathering result in a strong correlation between global weathering and TLF, while hydrology-dependent weathering results in a strong correlation with continental fragmentation (see Supplementary Information Section A.3, Supplementary Fig. 3).

### Analysis of reconstructed geologic data

Positive correlations between silicate weathering and TLF or fragmentation index, respectively, would imply, all else being equal, anti-correlations between global temperature and TLF or fragmentation index. This is because with a given CO_2_ outgassing rate, an increase in global weathering should cause a drawdown in CO_2_ and a corresponding reduction in temperature until the weathering rate is reduced back to equilibrium with outgassing. Further, the GCM/weathering results imply large differences between weathering regimes controlled by temperature and hydrology in terms of their responses to changes in TLF or changes in continental fragmentation. Therefore, by comparing proxy data for the evolution of global mean surface temperature (GMST) with reconstructed global continental geometry during the Phanerozoic and combining this information with the results from our GCM/weathering experiments, we can use the geologic record to gain insight into the relative strength of temperature vs. hydrology in stabilizing Earth’s climate through time.

To allow for the possibility that the Earth system may have shifted between hydrologically-controlled and temperature-controlled weathering at different times, we calculate the correlation coefficients across all time spans from 485 million years ago (Ma) to the present between a recent data-assimilation-based reconstruction of the Phanerozoic GMST record^47^ and the TLF and fragmentation index from a recent tectonic reconstruction of exposed land across the Phanerozoic^48^ using GPlates^112^. We converted the ShapeFiles from GPlates into NetCDF4 files encoding the exposed land from the^48^ reconstructions at every 10 Myr interval from 480 Ma to present on a latitude-longitude grid resolution of 1^∘^ by 1^∘^. Fragmentation index (total perimeter of exposed land divided by total area) only accounts for the outer boundary of land masses to avoid counting the boundaries of interior lakes and seas.

These calculations help differentiate between the competing hypotheses about the silicate weathering feedback described above by quantifying the statistical relationships between reconstructions of Earth’s paleogeography and surface temperature. See Supplementary Information Section A.1 for more detailed description of the correlation calculations.

## Supplementary information

Supplementary Information

Transparent Peer Review file

## Data Availability

Data and code for the paper are available on Zenodo at https://doi.org/10.5281/zenodo.16732630. The GCM simulations are also available at Baum and Fu^102^ (https://doi.org/10.5281/zenodo.6540321).

## Code availability

Code for the paper is available on Zenodo at https://doi.org/10.5281/zenodo.16732630.
