# Supplementary material for: Paleogeographic controls on Phanerozoic weathering rates and implications for climate regulation
Source: Nat Commun. 2026 Sep 3;17:10466. doi: 10.1038/s41467-026-77289-3 (PMC13635242; doi:10.1038/s41467-026-77289-3)
Supplement: Supplementary file 1 [file 41467_2026_77289_MOESM1_ESM.pdf]

# Supplementary Information for "Paleogeographic controls on Phanerozoic weathering rates and implications for climate regulation"

R.J. Graham[1*], B. B. Cael[1], M. Fu[2], D.S. Abbot[1]

[1]Department of Geophysical Sciences, University of Chicago, 5734 S. Ellis Avenue, Chicago, 60637, IL, USA.
[2]School of Ocean and Earth Science, University of Southampton, European Way, Southampton, SO14 3ZH, UK.

*Corresponding author(s). E-mail(s): arejaygraham@uchicago.edu

Reference numbers in the Supplementary Information correspond to the Bibliography in the Main Text.

## A.1 Regressing GMST against TLF and fragmentation index

To infer whether temperature and/or hydrology control weathering over different portions of the Phanerozoic, we regress global-mean surface temperature (GMST) against tropical land fraction (TLF) and fragmentation index over all possible sub-intervals of the Phanerozoic. We first interpolate GMST from Judd et al. [47] to the timestep of TLF and fragmentation using modified Akima interpolation. We then define two timepoints $t_1$ and $t_2$ that yield three sub-intervals of the Phanerozoic, i.e., (480, $t_1$), ($t_1$, $t_2$), and ($t_2$, present). For all possible combinations of $t_1$ and $t_2$, we calculate the Pearson correlation coefficient of GMST against TLF and fragmentation over all three sub-intervals. Altogether this indicates over which sub-intervals of the Phanerozoic GMST was significantly related to TLF and/or fragmentation.

Supplementary Figure 1 shows the results of this exercise, displaying the values of $t_1$ and $t_2$ where GMST is significantly anti-correlated with TLF over 480-$t_1$ Ma, significantly anti-correlated with fragmentation index over $t_1$-$t_2$ Ma, and significantly anti-correlated with TLF over $t_1$-0 Ma (no significant positive correlations were found over any interval). Locations are colored according to the most positive (i.e., weakest) Pearson correlation coefficient of the three sub-intervals; only sub-intervals with correlations that are significant at the 2.5% level are colored in. The range of valid $t_1$ and $t_2$ values shown in Supplementary Figure 1 is used to generate the black error bars in Fig. 5 of the main text, while the light blue shading represents the interval with mean values of $t_1$ (295 Ma) and $t_2$ (200 Ma).

## A.2 Analysis with Scotese et al. paleogeography

To examine whether our results were dataset dependent, we calculated TLF and fragmentation index for an alternative paleogeographic reconstruction from Scotese and Wright [50]. In this case, rather than extracting information from GPlates files like we did with Marcilly

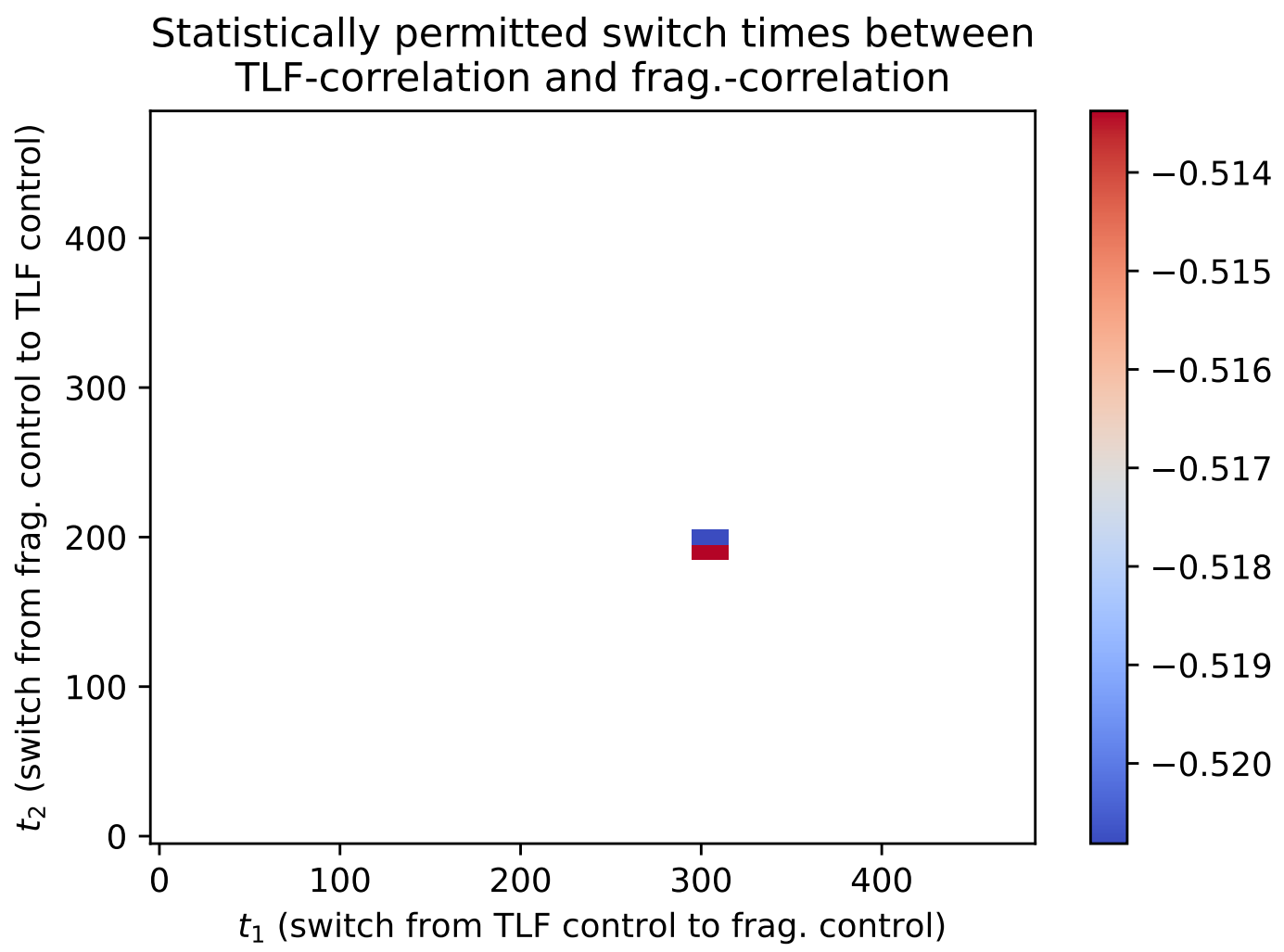


**Supplementary Figure 1 Statistically permitted times of switching between tropical land fraction (TLF) correlation and fragmentation correlation** Each grid point is colored according to the weakest Pearson correlation coefficient between global-mean surface temperature (GMST) and TLF or fragmentation across the three subintervals marked by that point. Colored points are significant at the 2.5% threshold across all three intervals, i.e. there is a significant TLF vs. GMST anti-correlation from 480 Ma to $t_1$.

et al. [48], we used CSV files with land elevation data provided by the PALEOMAP project [50] to reconstruct perimeter and TLF through time at 5 Myr intervals. The results are shown in Supplementary Figure 2, demonstrating that the same basic patterns apply to the Scotese and Wright [50] reconstructions, though the relationship between TLF and GMST is weaker between 0-200 Ma (see bottom left panel).

## A.3 MAC weathering

For comparison with the results in the main study with weathering calculated according to the WHAK [1] formulation, we also show that weathering calculated according to the Maher and Chamberlain [12] (MAC) demonstrates the same qualitative behavior by varying a parameter in the MAC model (path length over which water is in contact with weathering materials, which we will equate with soil depth for our purposes, though see supplement to Maher and Chamberlain [12] for discussion of situations in which the characteristic path length might be longer than soil depth) that moves the weathering regime from temperature-controlled to hydrology-controlled weathering (see Supplementary Figure 3). The MAC model accounts for the impact of clay formation in the weathering zone, which sets a maximum concentration on weathering products in runoff, drastically increasing the importance of hydrology for determining weathering fluxes when water moves through the weathering zone at a rate that allows for weathering products to reach the maximum chemically-equilibrated concentration. Larger soil depth increases the amount of time that weathering minerals are in contact with water, allowing weathering products to accumulate to higher concentrations and making it more likely the system will approach equilibrium between silicate dissolution and secondary mineral precipitation. When that chemical equilibrium is reached in the weathering zone, weathering is runoff limited since the concentrations of weathering products are

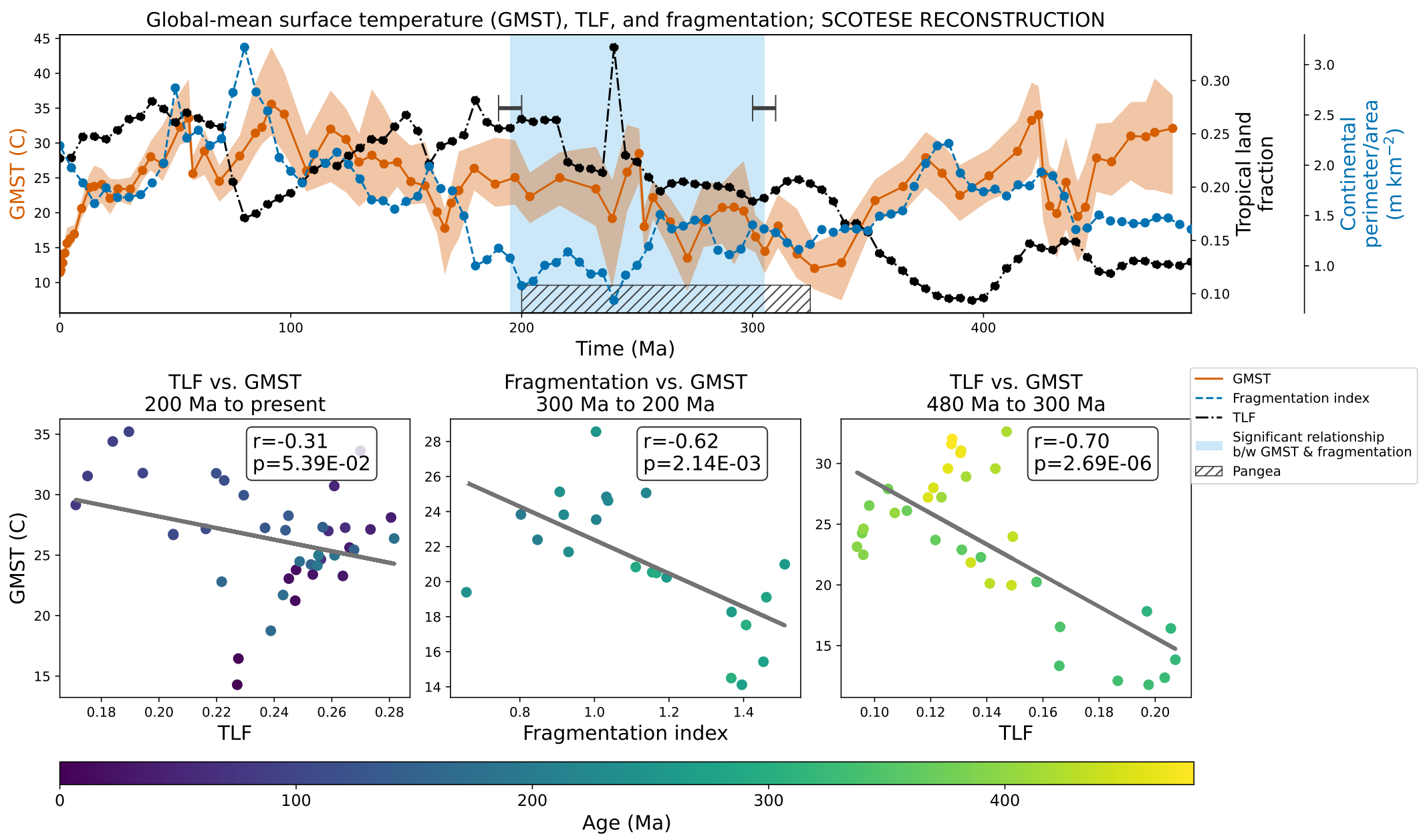


**Supplementary Figure 2  Time series of reconstructed global-mean surface temperature (GMST), tropical land fraction (TLF), and continental fragmentation (top panel) and scatter plots of GMST vs. TLF and fragmentation index (bottom panels) using paleogeography reconstructions from Scotese and Wright [50]**. In the top panel, GMST [47] is the red curve, TLF [50] is the black dash-dotted curve, and continental fragmentation is the dark blue dashed curve. The translucent red envelope represents the 95% confidence interval for GMST from Judd et al. [47]. The light blue region in the top panel represents the period continental fragmentation and GMST are significantly anti-correlated and the white regions are where TLF and GMST are significantly anti-correlated, with the error bars covering the range of times where the data allow for switches between the two modes. The hatched region covers the period during which Pangea existed [49]. The bottom three panels show correlation between GMST and (from left to right) TLF from 200 Ma to present, fragmentation index from 300 Ma to 200 Ma, and TLF again from 480 Ma to 300 Ma, with grey lines marking the lines of best fit, Pearson correlation coefficients in the upper right, and markers colored according to age.

capped, leading to hydrologic control. When the weathering zone is far from equilibrium, the kinetics of silicate dissolution determine the rate of weathering, leading to temperature control. Following Baum et al. [40]:

$$w = \frac{\alpha}{[k_{\mathrm{eff}}]^{-1} + mAt_{\mathrm{s}} + \alpha[qC_{\mathrm{eq}}]^{-1}}$$

where $w$ [mol m$^{-2}$ yr$^{-1}$] is the weathering flux from a given cell as in eqn. 2 of the main text; $\alpha = L\phi\rho_{\mathrm{sf}}AX_{\mathrm{r}}\mu$ is a parameter that combines weathering zone properties of characteristic water flow length scale ($L = 1$ m, often scaling with soil depth), porosity ($\phi = 0.1$), ratio of mineral mass to fluid volume ($\rho_{\mathrm{sf}} = 12728$ kg m$^{-3}$), and the mass fraction of minerals in the weathering zone that are weatherable ($X_{\mathrm{r}} = 0.36$); $k_{\mathrm{eff}} = k_{\mathrm{eff,ref}} \exp\left(\frac{T_{\mathrm{surf}} - T_{\mathrm{surf,ref}}}{T_{\mathrm{e}}}\right)\left(\frac{p\mathrm{CO}_2}{p\mathrm{CO}_{2,\mathrm{ref}}}\right)^{\beta}$ [mol m$^{-2}$ yr$^{-1}$] is the effective kinetic weathering rate, i.e., the weathering rate in the absence of chemical equilibration with clay formation, where the "ref" subscript denotes reference

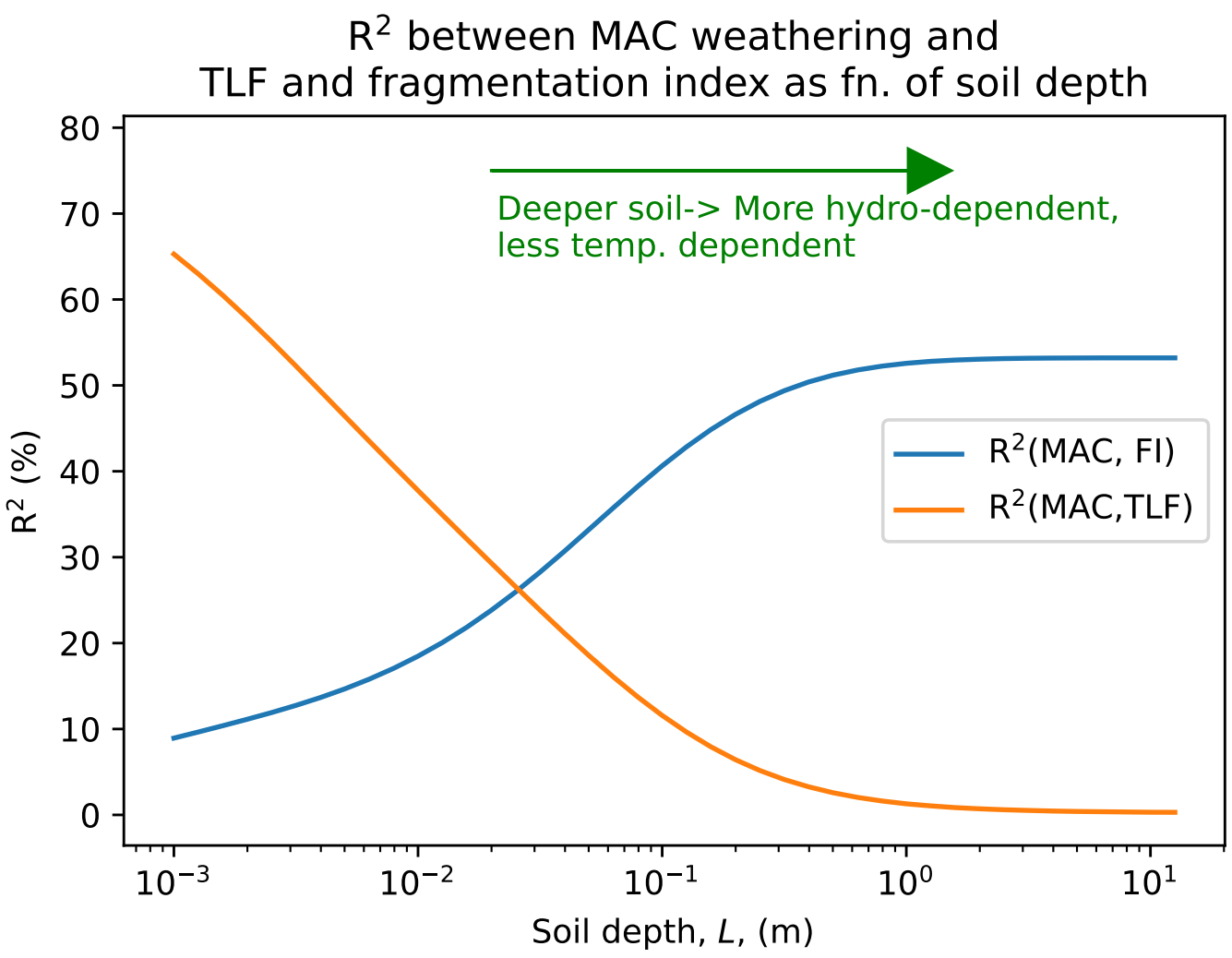


**Supplementary Figure 3 Coefficients of determination between Maher and Chamberlain [12] (MAC) silicate weathering and continental geometry parameters as a function of soil thickness.** The blue curve shows $R^2$ between MAC weathering and fragmentation index (FI). The orange curve shows $R^2$ between MAC weathering and tropical land fraction (TLF). The x-axis is soil thickness, with thick soils leading to runoff-limited weathering, i.e., hydrologic control, and thin soils leading to kinetically-limited weathering, i.e., temperature control.

values, $k_{\text{eff,ref}} = 8.7 \times 10^{-6}$ [mol m$^{-2}$ yr$^{-1}$], $T_{\text{surf,ref}}$=288.15 K, $T_e = 11.1$ K is the e-folding temperature, $pCO_{2,\text{ref}} = 280 \times 10^{-6}$ [bars], and $\beta = 0.3$ is the kinetic power-law $CO_2$ dependence; $m = 0.27$ [kg mol$^{-1}$] is the average molar mass of minerals being weathered; $A = 100$ [m$^2$ kg$^{-1}$] is the average specific surface area of the minerals being weathered; $t_s = 10^4$ [yr] is the mean age of the material being weathered; $q$ [m yr$^{-1}$] is the flux of water through the grid cell, given by the runoff from that grid cell output by the GCM; and $C_{\text{eq}} = \Lambda(pCO_2)^n$ [mol m$^{-3}$] is the maximum concentration of divalent cations in the water passing through weathering zones, as determined by chemical equilibrium between dissolving silicates and the secondary minerals (clays) that form from their products, with $\Lambda = 1.4 \times 10^{-3}$ as the thermodynamic coefficient and $n = 0.3$ as the thermodynamic power law $CO_2$ dependence (see Maher and Chamberlain [12], Graham and Pierrehumbert [19], Winnick and Maher [21] for more detailed explanations of these parameters).

## A.4 $CO_2$ sensitivity test

As noted in the main text (see Methods), we ran a smaller ensemble of GCM simulations with $CO_2$ concentrations of 500 ppm and 2000 ppm using a subset of 30 continental geometries out of the 120 configurations used with the 1000 ppm simulation in the main text (see Supplementary Figure 4). The 500 ppm and 2000 ppm ensembles show the same basic patterns of behavior with respect to changes in TLF and continental fragmentation (compare Fig. 4 of the main text with Supplementary Figure 4).

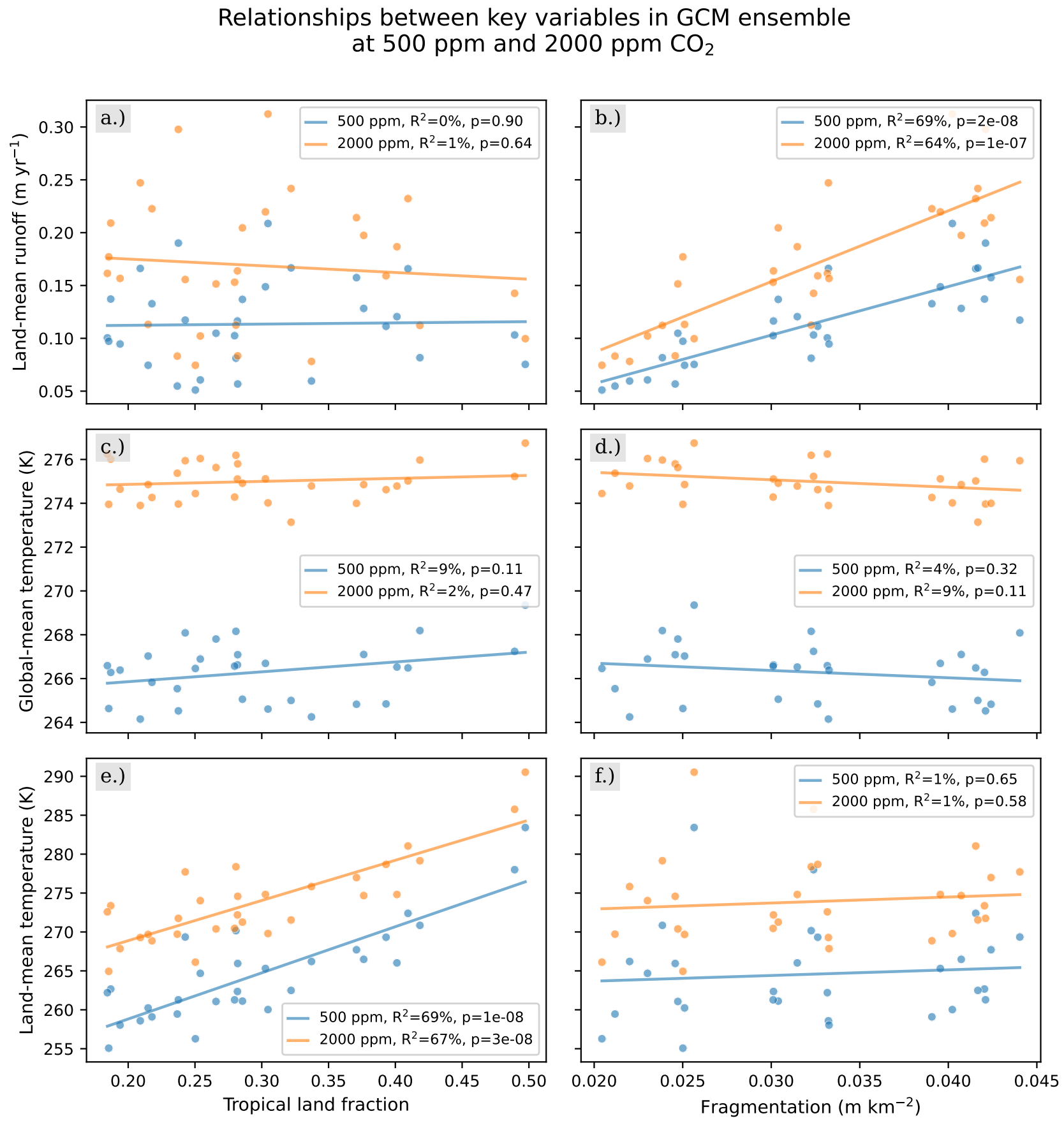


**Supplementary Figure 4 Scatter plots of average values of different variables from the global climate model (GCM) ensemble with $CO_2$ of 500 ppm (blue) and 2000 ppm (orange)** Panel a.) shows the lack of correlation between runoff and tropical land fraction (TLF); b.) shows a relatively strong correlation between runoff and fragmentation; c.) shows a weak correlation between global-mean surface temperature and TLF; d.) shows no correlation between global-mean surface temperature and fragmentation; e.) shows a strong correlation between global-mean land temperature and TLF; and f.) shows a lack of correlation between global-mean land temperature and fragmentation.
