# Supplementary material for: Paleogeographic controls on Phanerozoic weathering rates and implications for climate regulation
Source: Nat Commun. 2026 Sep 3;17:10466. doi: 10.1038/s41467-026-77289-3 (PMC13635242; doi:10.1038/s41467-026-77289-3)
Supplement: Supplementary file 2 [file 41467_2026_77289_MOESM2_ESM.pdf]

nature portfolio

Peer Review File

Paleogeographic controls on Phanerozoic weathering rates and implications for climate regulation

Corresponding Author: Dr R.J. Graham

This file contains all reviewer reports in order by version, followed by all author rebuttals in order by version.

Attachments originally included by the reviewers as part of their assessment can be found at the end of this file.

Version 0:

Reviewer comments:

Reviewer #1

(Remarks to the Author)
The manuscript 'Shifting mechanisms of climate stabilization across the Phanerozoic' by Graham et al. can be divided into two sections. First, the authors find "that when weathering is primarily temperature-controlled, global rates correlate strongly with tropical land fraction (TLF), whereas when it is primarily hydrologically controlled, rates correlate strongly with continental fragmentation" based on a series of CESM simulations. In the second part of the manuscript, the authors compare Phanerozoic changes in GMST to paleogeographic reconstructions to diagnose whether temperature or hydrology was a dominant factor in controlling global weathering rates, under the assumptions that GMST and CO2 are correlated and that weathering controls CO2.

The manuscript is well written and presents a clear narrative. It addresses a topic of major debate and uncertainty in paleoclimate science: the role of temperature versus hydrology in controlling weathering rates, and implications for the silicate weathering feedback as a thermostat. It raises several important points that are great scientific contributions, especially those related to the influence of paleogeography on the strength of temperature-controlled or runoff-controlled weathering. The use of idealized paleogeographies that cover a range of TLFs and land fragmentations is a neat way to draw generalized conclusions; the methodology is appropriate for the research aim. However, the extrapolation to what this implies for the Phanerozoic evolution is somewhat weak. I see great value in what the authors are trying to do and if (after some improvement) the relationships between CO2, GMST, and weathering can be further substantiated, the work could be very impactful and shed light on the long-lasting debate on whether temperature or hydrology is a more important control on weathering and how their contributions may have changed over time. This would require some extra work, however.

General comments

The first part of the manuscript demonstrates clearly that either fragmentation or TLF is important, depending on the dominant weathering rates control. These results are really interesting, and it would benefit from a more extended investigation into why this is. On Lines 138-150 reasons are given for why temperature-dependent weathering is mostly linked to TLF while runoff-dependent weathering is mostly linked to fragmentation. While straightforward, the reader needs to see the evidence to back this up. In current form, the reader cannot judge how temperature and runoff change with fragmentation and TLF. For example, I imagine that temperature and fragmentation are not entirely decoupled as fragmentation affects ocean-atmosphere interaction. Especially the statement "[…] changing TLF does not tend to lead to large changes in runoff, […]" needs further explanation; this is not per se intuitive. An increase in TLF can also increase runoff as more land is located in high-precipitation regions.

The following statement written near Line 152 leads into my next comment relevant for the second part of this manuscript:

"If Earth's carbonate-silicate thermostat has been dominated by surface weathering that is primarily sensitive to surface temperatures, we should observe an anticorrelation between TLF and surface temperature through time, because an increase in TLF would be expected to increase the weathering rate and cause CO2 drawdown and cooling and a reduction in TLF would slow weathering and allow CO2 to accumulate, leading to warming."

There are two important assumptions here that form the backbone to the conclusions drawn in the manuscript.

1. The authors assume a direct link between CO_2 and temperature which is perhaps somewhat simplistic. While it is true for most of the Phanerozoic, Judd et al. (2024) also point out that there is no strong correlation between CO_2 and GMST during the Mesozoic (250-66 Ma). Under the same CO_2, global mean temperatures can differ when the land mass configuration is different. Lands have a different heat capacity and albedo profile than oceans, and changes in heat distribution by ocean currents change with landmass. In addition, some paleogeographies are more prone to develop ice sheets with can lead to further decoupling of GMST and CO_2. This point needs to be addressed in my opinion to back up why GMST can be used to infer weathering regimes.
A major benefit of the idealized GCM ensemble is that you could identify the extent to which landmass distribution affects the global temperature (under fixed CO_2) and use that to discuss the implications for the Phanerozoic GMST-CO_2 relationships. Further, I am wondering about the role of background climate state. The Phanerozoic has seen large climate change. Paleoclimate proxy records suggest that at higher global temperatures, the latitudinal temperature gradient is less steep and hydrological cycling is more intense, presumably with consequences for the importance of TLF and fragmentation in controlling the weathering strengths?

2. The authors assume a direct link between (high) weathering and (low) CO_2. While intuitive, it may not always be the case. For instance, a recent paper (Hülse and Ridgwell, 2025) shows that CO_2 drawdown from silicate weathering may be supplemented by enhanced CO_2 drawdown from concurrent organic carbon burial, i.e. carbon burial is also important in controlling CO_2, especially during times of low oxygenation, and may destabilize the thermostat. Further, some geological intervals are characterized by high CO_2 as well as high weathering rates that can be sustained for millions of years because CO_2 emissions (e.g. volcanism) exceed the weathering rate.
Whether these processes are relevant for Phanerozoic intervals on the long-timescales studies here should at least be discussed, substantiated, or refuted.

All in all, the manuscript is incredibly interesting and could serve as a significant scientific contribution. I would very much like to see this work published, but in the current iteration, the uncertainties are not yet sufficiently addressed and are perhaps too large to justify the conclusion that the weathering regime changed throughout the Phanerozoic based solely on the correlation between landmass distribution and GMST. Nonetheless, I think there is potential for improvement. For instance, using the idealized GCM ensemble to quantify the relation between GMST and landmass. I would also like to highlight that a full series of Phanerozoic GCM (HadCM3) simulations exists (Valdes et al., 2020) with published climatological fields of runoff and temperate that may be interesting to consider in this study.

Minor comments

- Caption Fig. 1&2. The caption reads
"Left column: Continental configuration with high fragmentation index ($p = -1$, dispersed continents). Middle column: Configuration with medium fragmentation index ($p = -2$, partially fragmented). Right column: Configuration with small fragmentation index ($p = -3$, supercontinental)."
I believe the right and left columns are swapped so it should indicate that the left columns have a small fragmentation index (supercontinental) and the right columns have high fragmentation index (dispersed continents). Also unclear what the values $p=-1$ to $p=-3$ stand for, add reference to Methods or explain.
- Line 132. Define k and α in text.
- Line 133. Define Wtot in text.
- Line 299. Remove degree sign for eccentricity (it's a unitless metric)
- Fig 4. I'm struggling to read the yellow y-label. Choose different color?

References
Valdes, P.J., Scotese, C.R. and Lunt, D.J., 2020. Deep ocean temperatures through time. Climate of the Past Discussions, 2020, pp.1-37.
Hülse, D. and Ridgwell, A., 2025. Instability in the geological regulation of Earth's climate. Science, 389(6767), p.eadh7730.

(Remarks on code availability)

Reviewer #2

(Remarks to the Author)
Comment on "Shifting mechanisms of climate stabilization across the Phanerozoic" by Graham et al.

Summary: This work used "tropical land fraction (TLF)" and "continental fragmentation" as proxies for temperature / hydrological controlled-weathering, respectively, and applied them in geological records (i.e., Temperature). The manuscript is well written with clear logic flow and comprehensive figures. Novelty is intermediate to high. There are some points I would like the authors to explain more clearly. Overall, I would recommend a minor revision.

Main point:
1) The author used a simplified weathering equation to demonstrate: if they vary hydrology parameter, the weathering fluxes would increase as continental fragmentation (CF) proxy increase. Accordingly, varying temperature parameter in the

equation, the weathering fluxes would also increases as TLF proxy increases. This makes sense if we assume the equation is reasonable. However, in later part of the work, the authors then use CF and TLF as proxies for hydrological/temperature controlled weathering (i.e., reverse demonstrating). This does not make sense, because your relationship between CF/TLF proxy and the hydrological/temp controlled-weathering is sufficient but not compulsory. That is to say, changing alpha or k -> changing CF/TLF-influenced weathering, but changing CF/TLF-influenced weathering does not solely -> changing alpha or k, because other factors may also influencing CF/TLF-derived weathering (e.g., CO2 level-induced dissolution rate change, tectonics, lithology change, plants colonization, monsoon, etc.), and the authors have roughly acknowledge this point at L237-254). If the paper just said (based on the data), continental configuration influenced global temperature, I would agree.
2) In geological record part, correlation does not mean causality (Fig 4).
3) In geological timescale, CO2 release (weathering of organic carbon + degassing) = CO2 removal (mainly silicate weathering) (as authors already suggested). Many studies used a term weathering sensitivity to understand the weathering process in this context (under certain CO2 level, how much weathering fluxes could be produced). The equation looks like: W = k * (RCO2)^a, where W is weathering flux, k is weatherability, RCO2 is CO2 ratio to modern level, and a is a fitting parameter (e.g., see Penmann et al., 2020, ESR). In my eyes, what the authors trying to do in this manuscript is actually to understand what's inside the weatherability term k. If so, personally, I think it would be great if authors could compare the work with previous weatherability works (e.g., Caves Rugenstein et al., 2019 Nature, etc.).

Line-by-line comment:
This manuscript is well written and thus I only have very little L-by-L comments…
Method: I remembered Josh West 2012 (Geology) used slightly different version of hydrological term in weathering equation. Can you make a comparison how they behave differently?
L210-214: The timescale in this manuscript is multi-Ma, and I guess it is way longer than 'future lifespan' timescale?
L217-223: sorry I did not get the logic behind this sentence.
L328: if I understood it right, Wref are different in CF and TLF scenarios. Are the results still comparable?
The choice of weathering equation: can I ask why using power law for hydrological control?
Also, can I ask where is the model validation (the Baum et al paper is the validation for your work?)?
L332: therefore, terrestrial reverse weathering is not considered?
L474: there is a figure in reference?
Finally: I failed to access to the Zenodo link provided. Is it possible to provide a GitHub link?

(Remarks on code availability)
I am not able to access to the zenodo repository to review the code (i.e., zenodo says the link is broken). I kindly ask whether the authors could provide the github link instead of zenodo (might work better)

Reviewer #3

(Remarks to the Author)
Review of « Shifting mechanisms of climate stabilization across the Phanerozoic » (NCOMMS-25-86427) by R.J. Graham et al.

SUMMARY
Graham et al. analyze the drivers of continental weathering during the Phanerozoic. They first analyze a series of 120 previously-published climatic simulations conducted on idealized continental configurations with a relatively low-resolution version of CESM. They consider temperature-only and runoff-only representations of weathering on land, and demonstrate that total weathering is driven by the low-latitude land area and continental fragmentation, respectively. In a second step, based on these lessons, the authors compare geological reconstructions of global surface air temperature, low-latitude land area and continental fragmentation during the Phanerozoic, in order to identify the periods of time during which continental weathering was driven more by temperature or more by runoff. The authors conclude that temperature was the main driver of continental weathering during the Phanerozoic, with runoff only being the key player during the 300–200 Ma time interval.

GENERAL
I really appreciated the manuscript by Graham et al. It proposes an original approach based on reasonable methods. It is very well written and the figures are of good quality. I support the publication of the manuscript after revisions. Please note that my expertise in weathering is limited; hence I mostly review the general aspect of the manuscript and suggest to contact an expert in weathering for complementary comments.

MAJOR POINTS

- The results largely rely on the geological datasets used. While I acknowledge the large margins of uncertainty associated with climatic reconstructions (see for instance Grossman and Joachimski, 2022), I think that the one of Judd et al. (2024) is a very good choice. Regarding the paleogeographical reconstructions, the authors decided to use the ones of Marcilly et al. (2021). Considering that several alternative Phanerozoic-scale paleogeographical reconstructions are now available in numerical format, I would like to see a sensitivity test that would permit determining the degree of dependence of the conclusions to the dataset used. I suggest to test the reconstructions of the PALEOMAP project (Scotese and Wright, 2018). They are widely used in the deep-time community. Including these additional calculations would be of great interest to the general readership. These maps are also available every 5 Myrs, which would provide more data points for correlations / statistical tests.

- Lines 151–164: this rationale supposes an invariant volcanic outgassing rate, i.e., that global temperature is controlled only

by the CO2 sink with no substantial contribution of the source. This strong assumption is discussed on lines 263–268; considering its first-order impact, I suggest to state that up front. In particular, fragmentation and volcanism are probably closely related, with periods of continental fragmentation (hence mid-ocean ridge activity) probably associated with strong volcanic outgassing rates? If so, a strong fragmentation would be associated with high volcanic outgassing rates hence presumably warmer climates in the paleoclimate reconstructions. I think the results are thus biased towards an underestimation of the impact of the hydrological cycle, which may explain why only 20 % of the time interval is found to be associated with a runoff-dominated weathering regime? I think this point deserves some in-depth discussion.

SECONDARY POINTS

- I had to read the Methods and have a look at Baum et al. (2022) at the start of section "Sensitivity of silicate weathering to tropical land fraction…". I think it is important to include a short summary of the key elements here to make the manuscript accessible to a reader with no previous knowledge of Baum et al. (2022). In particular, the fact that the 120 continental configurations are idealized was at first a bit surprising for me; I suggest to introduce such key notions here, as well as key notions regarding the calculations of weathering and the fact that simulations were conducted using a constant pCO2, etc. Actually, it may help to state as early as in the abstract, that the continental configurations are idealized.

- Lines 123–125: the authors state that "hydrological controlled weathering […] is not strongly affected by TLF". I don't think this is exact; TLF can induce a doubling of weathering (from 1.8 to 4.0), close to the impact of fragmentation. I think the key point here is that weathering does not change monotically with TLF.

- I think that the authors may also want to discuss the potential modulation of weathering by reverse weathering in their discussion (e.g., Isson and Rauzi, 2024).

- From Fig. 4, it seems that the interval 485 to ~420 Ma may rather be hydrology-controlled? At least, weathering at that time does not seem to be temperature-controlled, based on the apparent co-variation of GMST and TLF, after the top and bottom-right panels. Excluding these time slices from the correlation may increase the p-value (of 0.025 after line 172?). Similarly, I guess one could discuss the controls of weathering over the last ~25 Myrs after Fig. 4.

TECHNICAL COMMENTS
- Line 32: a period is missing.
- Lines 83–88: please consider splitting the very long sentence.
- Lines 245–248: the authors may consider citing also the mechanistic modeling approach of Maffre et al. (2022).
- Caption of Fig. 1: the description of high/low fragmentation index is reversed.
- Caption of Fig. 4: missing space in first line.
- Fig. 4: need to add some space on the right of "tropical land fraction" to avoid overlapping with the 3rd y-axis. + I suggest to add p-values to bottom panels.
- Please remind the reader where GCM fields can be publicly accessed for convenience.

Best regards,
Alexandre Pohl (CNRS, France)

REFERENCES CITED
Baum, M., Fu, M., Bourguet, S., 2022. Sensitive Dependence of Global Climate to Continental Geometry. Geophysical Research Letters 49, 1–8. https://doi.org/10.1029/2022gl098843
Goddéris, Y., Donnadieu, Y., Carretier, S., Aretz, M., Dera, G., MacOuin, M., Regard, V., 2017. Onset and ending of the late Palaeozoic ice age triggered by tectonically paced rock weathering. Nature Geoscience 10, 382–386. https://doi.org/10.1038/ngeo2931
Grossman, E.L., Joachimski, M.M., 2022. Ocean temperatures through the Phanerozoic reassessed. Scientific Reports 1–14. https://doi.org/10.1038/s41598-022-11493-1
Isson, T., Rauzi, S., 2024. Oxygen isotope ensemble reveals Earth's seawater, temperature, and carbon cycle history. Science 383, 666–670. https://doi.org/10.1126/science.adg1366
Judd, E.J., Tierney, J.E., Lunt, D.J., Montañez, I.P., Huber, B.T., Wing, S.L., Valdes, P.J., 2024. A 485-million-year history of Earth's surface temperature. Science 385, eadk3705. https://doi.org/10.1126/science.adk3705
Maffre, P., Chiang, J.C.H., Swanson-Hysell, N.L., 2023. The effect of the Pliocene temperature pattern on silicate weathering and Pliocene–Pleistocene cooling. Clim. Past 19, 1461–1479. https://doi.org/10.5194/cp-19-1461-2023
Maffre, P., Goddéris, Y., Le Hir, G., Nardin, É., Sarr, A.-C., Donnadieu, Y., 2025. GEOCLIM7, an Earth system model for multi-million-year evolution of the geochemical cycles and climate. Geosci. Model Dev. 18, 6367–6413. https://doi.org/10.5194/gmd-18-6367-2025
Maffre, P., Goddéris, Y., Pohl, A., Donnadieu, Y., Carretier, S., Le Hir, G., 2022. The complex response of continental silicate rock weathering to the colonization of the continents by vascular plants in the Devonian. American Journal of Science 322, 461–492. https://doi.org/10.2475/03.2022.02
Marcilly, C.M., Torsvik, T.H., Domeier, M., Royer, D.L., 2021. New paleogeographic and degassing parameters for long-term carbon cycle models. Gondwana Research. https://doi.org/10.1016/j.gr.2021.05.016
Martin, P.E., Macdonald, F.A., McQuarrie, N., Flowers, R.M., Maffre, P.J.Y., 2023. The rise of New Guinea and the fall of Neogene global temperatures. Proc. Natl. Acad. Sci. U.S.A. 120, e2306492120. https://doi.org/10.1073/pnas.2306492120

(Remarks on code availability)
Permission required
"You do not have sufficient permissions to view this page."

Version 1:

Reviewer comments:

Reviewer #1

(Remarks to the Author)
See review file attached.

(Remarks on code availability)
The link to the repository works. All files seem to be present, including an extensive README. I did not try to run the Phyton code but, based on visual inspection of the scripts, the code looks clear and understandable.

Reviewer #2

(Remarks to the Author)
Second round of comment on “Paleogeographic controls on Phanerozoic weathering rates and implications for climate regulation”.

It's great to see the authors have made a detailed reply and according changes in the revised manuscript. Major points (from the 1st round comment) have been resolved and discussed with details (L243-263, L264-282). Influences of choosing the power-law style equation to overall results have been discussed. The code is also available right now. From my side, this manuscript is well-written.
Few points:
1. Remind readers, GMST is a proxy for CO2 and ultimately the proxy for weathering flux at each timestep.
2. Two things from my-side of unclarity from the 1st round of comment. 1) What I mean ‘terrestrial reverse weathering’ is secondary mineral formation on land. This is normally called ‘weathering congruency’. If congruency is low, a great share of dissolved phases would be precipitated as sec. min. before draining into the ocean, and apparently this would change the weathering flux. 2) What I mean about model validation is the choice of the silicate weathering equations and parameters have been demonstrated with observed data/experimental data.
3. From L372: I think the authors have introduced the silicate weathering equation nicely in the ‘reply’, but not in the ‘Method’ part of the manuscript. I would recommend authors briefly introduce to the audience the reasons for choosing this equation.

(Remarks on code availability)

Reviewer #3

(Remarks to the Author)
The authors satisfactorily addressed my previous comments.
This is an excellent manuscript, which I think is now read for publication.
– Alexandre Pohl (CNRS, France)

(Remarks on code availability)

Version 2:

Reviewer comments:

Reviewer #1

(Remarks to the Author)
I am satisfied with the revisions to the manuscript. The authors have meticulously addressed the comments that myself and other reviewers raised. The study is incredibly interesting in that the authors use an original approach to study what physical processes contributed to the large-scale evolution of Earth’s climate across time. I do not have further comments and recommend publication as is.

Pam Vervoort

(Remarks on code availability)

Reviewer #2

(Remarks to the Author)
After reading author's responses, I am satisfied with the logic and contents of the manuscript. I think it is now the time for the audience to read and assess the manuscript.

(Remarks on code availability)

Open Access This Peer Review File is licensed under a Creative Commons Attribution 4.0 International License, which permits use, sharing, adaptation, distribution and reproduction in any medium or format, as long as you give appropriate credit to the original author(s) and the source, provide a link to the Creative Commons license, and indicate if changes were made.
In cases where reviewers are anonymous, credit should be given to 'Anonymous Referee' and the source.
The images or other third party material in this Peer Review File are included in the article’s Creative Commons license, unless indicated otherwise in a credit line to the material. If material is not included in the article’s Creative Commons license and your intended use is not permitted by statutory regulation or exceeds the permitted use, you will need to obtain permission directly from the copyright holder.
To view a copy of this license, visit https://creativecommons.org/licenses/by/4.0/

Reviewer Comments and Author Responses

"Shifting mechanisms of climate stabilization across the Phanerozoic" (now "Paleogeographic controls on Phanerozoic weathering rates and implications for climate regulation") by Graham et al.

Contents

1 Reviewer #1 (Remarks to the Author) 2
1.1 General Comments 2
1.2 Minor Comments 5
1.3 References Cited by Reviewer #1 6

2 Reviewer #2 (Remarks to the Author) 6
2.1 Main Points 6
2.2 Line-by-Line Comments 8
2.3 Reviewer #2 (Remarks on Code Availability) 11

3 Reviewer #3 (Remarks to the Author) 11
3.1 Summary 11
3.2 General 11
3.3 Major Points 11
3.4 Secondary Points 13
3.5 Technical Comments 14
3.6 Reviewer #3 (Remarks on Code Availability) 15
3.7 References Cited by Reviewer 3 15

1 Reviewer #1 (Remarks to the Author)

The manuscript 'Shifting mechanisms of climate stabilization across the Phanerozoic' by Graham et al. can be divided into two sections. First, the authors find "that when weathering is primarily temperature-controlled, global rates correlate strongly with tropical land fraction (TLF), whereas when it is primarily hydrologically controlled, rates correlate strongly with continental fragmentation" based on a series of CESM simulations. In the second part of the manuscript, the authors compare Phanerozoic changes in GMST to paleogeographic reconstructions to diagnose whether temperature or hydrology was a dominant factor in controlling global weathering rates, under the assumptions that GMST and CO_2 are correlated and that weathering controls CO_2.

The manuscript is well written and presents a clear narrative. It addresses a topic of major debate and uncertainty in paleoclimate science: the role of temperature versus hydrology in controlling weathering rates, and implications for the silicate weathering feedback as a thermostat. It raises several important points that are great scientific contributions, especially those related to the influence of paleogeography on the strength of temperature-controlled or runoff-controlled weathering. The use of idealized paleogeographies that cover a range of TLFs and land fragmentations is a neat way to draw generalized conclusions; the methodology is appropriate for the research aim. However, the extrapolation to what this implies for the Phanerozoic evolution is somewhat weak. I see great value in what the authors are trying to do and if (after some improvement) the relationships between CO_2, GMST, and weathering can be further substantiated, the work could be very impactful and shed light on the long-lasting debate on whether temperature or hydrology is a more important control on weathering and how their contributions may have changed over time. This would require some extra work, however.

> **Author response:** We appreciate your positive words and your productive suggestions.

1.1 General Comments

The first part of the manuscript demonstrates clearly that either fragmentation or TLF is important, depending on the dominant weathering rates control. These results are really interesting, and it would benefit from a more extended investigation into why this is. On Lines 138–150 reasons are given for why temperature-dependent weathering is mostly linked to TLF while runoff-dependent weathering is mostly linked to fragmentation. While straightforward, the reader needs to see the evidence to back this up. In current form, the reader cannot judge how temperature and runoff change with fragmentation and TLF. For example, I imagine that temperature and fragmentation are not entirely decoupled as fragmentation affects ocean-atmosphere interaction. Especially the statement "[. . .] changing TLF does not tend to lead to large changes in runoff, [. . .]" needs further explanation; this is not per se intuitive. An increase in TLF can also increase runoff as more land is located in high-precipitation regions.

> **Author response:** We have added a new figure (figure 4) showing how runoff, global-mean surface temperature, and global-mean land temperature all vary with TLF and continental fragmentation. We altered lines 152 to 171 to read:
>
> The reason for this divergence in response to continental geometry is straightforward. Within the GCM ensemble, global runoff is uncorrelated with TLF (R^2 = 0%) but strongly correlated with continental fragmentation (R^2 = 54%; see Fig. 4a, b). Therefore, when weathering is dominated by hydrology, changes to TLF have a minimal effect,

but changes to fragmentation can alter weathering rates through their impact on runoff, with more fragmented configurations experiencing active rainfall and runoff over a larger fraction of their land. Conversely, although global-mean surface temperature is both barely correlated with TLF ($R^2 = 16\%$) and uncorrelated with continental fragmentation ($R^2 = 0\%$; see Fig. 4c, d), global-mean *land* temperature is strongly correlated with TLF ($R^2 = 71\%$), while remaining uncorrelated with fragmentation ($R^2 = 1\%$; see Fig. 4e, f). So, when temperature is the main driver of weathering, changes to TLF can change the weathering rate by altering the amount of land that experiences higher temperatures near the equator. The small positive relationship between global-mean surface temperature and TLF visible in Fig. 4c is due to a combination of the reduction of high-albedo clouds when tropical ocean is replaced with mostly cloud-free land in high-TLF simulations, as well as an increase in high-albedo land snow in low-TLF simulations where land is colder and closer to the poles. Inclusion of ocean heat transport could modulate this effect by redistributing energy from the tropics toward the poles, reducing or eliminating the correlation entirely Jenkins [1995].

The following statement written near Line 152 leads into my next comment relevant for the second part of this manuscript:

"If Earth's carbonate-silicate thermostat has been dominated by surface weathering that is primarily sensitive to surface temperatures, we should observe an anticorrelation between TLF and surface temperature through time, because an increase in TLF would be expected to increase the weathering rate and cause CO_2 drawdown and cooling and a reduction in TLF would slow weathering and allow CO_2 to accumulate, leading to warming."

There are two important assumptions here that form the backbone to the conclusions drawn in the manuscript.

1. The authors assume a direct link between CO_2 and temperature which is perhaps somewhat simplistic. While it is true for most of the Phanerozoic, Judd et al. (2024) also point out that there is no strong correlation between CO_2 and GMST during the Mesozoic (250–66 Ma). Under the same CO_2, global mean temperatures can differ when the land mass configuration is different. Lands have a different heat capacity and albedo profile than oceans, and changes in heat distribution by ocean currents change with landmass. In addition, some paleogeographies are more prone to develop ice sheets with can lead to further decoupling of GMST and CO_2. This point needs to be addressed in my opinion to back up why GMST can be used to infer weathering regimes. A major benefit of the idealized GCM ensemble is that you could identify the extent to which landmass distribution affects the global temperature (under fixed CO_2) and use that to discuss the implications for the Phanerozoic GMST–CO_2 relationships.

Author response: In addition to the addition of Figure 4 and discussion of the relationships between temperature, TLF, and fragmentation noted above, we have now added the following to lines 235 to 242:

In any case, the reconstructed GMST of Judd et al. [2024] correlates strongly with (log-transformed) reconstructed CO_2 over most of the Phanerozoic [$r = 0.77$, $p < 0.01$, Judd et al., 2024], and our GCM ensemble displayed weak or non-existent relationships between global-mean surface temperature and both TLF and continental fragmentation (Fig. 4c, d). This increases our confidence that it is a reasonable first-order assumption that CO_2 and temperature were tightly linked over most of the Phanerozoic and that

variations in continental geometry were unlikely to have a large direct (i.e., non-carbon-cycle-mediated) effect on surface temperatures.

Further, I am wondering about the role of background climate state. The Phanerozoic has seen large climate change. Paleoclimate proxy records suggest that at higher global temperatures, the latitudinal temperature gradient is less steep and hydrological cycling is more intense, presumably with consequences for the importance of TLF and fragmentation in controlling the weathering strengths?

Author response: Latitudinal temperature gradient has varied by a bit less than a factor of two over the past 95 million years, which encompasses the hottest period of the Phanerozoic, corresponding to a minimum equator-to-pole temperature gradient of 15.6 C (Gaskell et al., 2022). If we assume a fairly weak temperature feedback with an e-folding temperature of 30 C (corresponding to k = 1/30 C-1), then teleporting a continent from pole to equator would multiply weathering by a factor of $\exp \frac{15.6}{30} \approx 1.7$. With a stronger e-folding temperature of 15 C (k=1/15 C-1), movement from pole to equator would increase weathering by a factor $\exp \frac{15.6}{15} \approx 5.9$. In other words, with the weakest expected equator-to-pole temperature gradient, TLF should still significantly impact weathering rates even if the temperature feedback is weak, and if the temperature feedback is strong, the effect can still be quite large.

Regarding changes to hydrological cycling, if weathering is primarily temperature-dependent, then more intense hydrological cycling won't have much impact on weathering. If weathering is primarily hydrologically-mediated, then more intense hydrological cycling will lead to increased weathering, since that is the basic feedback mechanism. Unless the background temperature state is somehow influencing the feedback mechanism, it shouldn't affect the point that fragmentation is important for hydrologically-controlled weathering but not for temperature-controlled weathering. In a set of calculations not included in the manuscript, we looked into this a bit using a subset of the idealized paleogeographies in the paper (just 10 instead of 40), evaluating the correlations at 500 ppm and 2000 ppm (vs. 1000 ppm in the paper); the same bulk correlations appear.

2. The authors assume a direct link between (high) weathering and (low) CO_2. While intuitive, it may not always be the case. For instance, a recent paper (Hülse and Ridgwell, 2025) shows that CO_2 drawdown from silicate weathering may be supplemented by enhanced CO_2 drawdown from concurrent organic carbon burial, i.e. carbon burial is also important in controlling CO_2, especially during times of low oxygenation, and may destabilize the thermostat. Further, some geological intervals are characterized by high CO_2 as well as high weathering rates that can be sustained for millions of years because CO_2 emissions (e.g. volcanism) exceed the weathering rate.

Whether these processes are relevant for Phanerozoic intervals on the long-timescales studies here should at least be discussed, substantiated, or refuted.

Author response: This point, while well-taken, is a fundamental question deserving of its own study. To briefly address why we think the assumption of equality between degassing and silicate weathering is reasonable for our study: The Hülse and Ridgwell paper only carries simulations out to 500,000 years, and based on Fig. 3 in their paper it appears that regardless of the level of transient cooling from the carbon cycle instability, the CO_2 values are converging back to the equilibrium level that existed before the imposed climate perturbation. It doesn't appear that the instability suggested in the paper actually impacts the long-term equilibrium state of the atmosphere unless the

climate hits a bifurcation point and globally glaciates during the transient cooling, and the transient cooling is maximized at lower oxygen values than we have seen over most of the Phanerozoic. Re: the point about CO_2 emissions exceeding weathering for millions of years, this is theoretically possible but it would lead to very large excursions in climate that would likely eliminate complex life, see e.g., Berner and Caldeira, 1997; Broecker and Sanyal, 1998; or Zeebe and Caldeira, 2008.

That said, we have added a brief mention of these issues. Lines 52 to 59 now read:

Although transient imbalances between CO_2 inputs and outputs due to organic carbon interactions [Hülse and Ridgwell, 2025] or limitations in silicate mineral supply [Kump, 2018] may have played important roles in Earth's climate evolution, weathering-based coupling between atmospheric CO_2 removal and background climate appears to play a key role in maintaining the quasi-equilibrium in the carbon cycle necessary to avoid frequent excursions to extremely high or low surface temperatures that would have threatened the existence of complex life on Earth during the Phanerozoic [Broecker and Sanyal, 1998, Zeebe and Caldeira, 2008]

All in all, the manuscript is incredibly interesting and could serve as a significant scientific contribution. I would very much like to see this work published, but in the current iteration, the uncertainties are not yet sufficiently addressed and are perhaps too large to justify the conclusion that the weathering regime changed throughout the Phanerozoic based solely on the correlation between landmass distribution and GMST. Nonetheless, I think there is potential for improvement. For instance, using the idealized GCM ensemble to quantify the relation between GMST and landmass. I would also like to highlight that a full series of Phanerozoic GCM (HadCM3) simulations exists (Valdes et al., 2020) with published climatological fields of runoff and temperate that may be interesting to consider in this study.

Author response: We appreciate the positive and constructive comments. We quantified the relationship between GMST and landmass using the GCM results like you suggested. We elected not to analyze the Valdes dataset in this study; however it is a point we have noted for follow-up, and we appreciate it.

1.2 Minor Comments

- Caption Fig. 1 & 2. The caption reads: "Left column: Continental configuration with high fragmentation index ($p = -1$, dispersed continents). Middle column: Configuration with medium fragmentation index ($p = -2$, partially fragmented). Right column: Configuration with small fragmentation index ($p = -3$, supercontinental)." I believe the right and left columns are swapped so it should indicate that the left columns have a small fragmentation index (supercontinental) and the right columns have high fragmentation index (dispersed continents). Also unclear what the values $p = -1$ to $p = -3$ stand for, add reference to Methods or explain.

  Author response: You're right, we flipped the order of the columns in the figure without changing the caption to match, thank you for the reminder. That is now fixed. And the "$p = -1, -2, -3$" values correspond to the p-exponent in equation (1), i.e., the slope or color of the power spectrum of the continental geometry. We removed the reference to p in the captions since it is not particularly important for understanding.

- Line 132. Define k and α in text.

Author response: Lines 140 through 144 now read:

To quantify these apparent relationships between weathering and continental geometry, we calculated coefficients of determination between the global weathering rate and TLF and continental fragmentation for our GCM ensemble over a wide range of values for k (temperature-dependence of weathering; see eqn. 1 in **Methods**) and α (runoff-dependence of weathering; see eqn. 1), with results shown in Fig. 3.

- Line 133. Define W_{tot} in text.

  Author response: Lines 144 through 149 now read:

  When the weathering feedback is dominated by hydrology instead of temperature (large α, small k), the coefficient of determination between the total global weathering rate (W_{tot}) and the fragmentation index, $\mathrm{R}^2(W_{\mathrm{tot}}$, fragmentation index), can exceed 50% (bottom right corner of top panel in Fig. 3), while $\mathrm{R}^2(W_{\mathrm{tot}}$, TLF) falls to nearly zero (bottom right corner of bottom panel in Fig. 3).

- Line 299. Remove degree sign for eccentricity (it's a unitless metric).

  Author response: Done.

- Fig 4. I'm struggling to read the yellow y-label. Choose different color?

  Author response: We have changed it to a darker color.

1.3 References Cited by Reviewer #1

- Valdes, P.J., Scotese, C.R. and Lunt, D.J., 2020. Deep ocean temperatures through time. *Climate of the Past Discussions*, 2020, pp. 1–37.
- Hülse, D. and Ridgwell, A., 2025. Instability in the geological regulation of Earth's climate. *Science*, 389(6767), p. eadh7730.

Best regards, Alexandre Pohl (CNRS, France)

2 Reviewer #2 (Remarks to the Author)

Comment on "Shifting mechanisms of climate stabilization across the Phanerozoic" by Graham et al.

Summary: This work used "tropical land fraction (TLF)" and "continental fragmentation" as proxies for temperature / hydrological controlled-weathering, respectively, and applied them in geological records (i.e., Temperature). The manuscript is well written with clear logic flow and comprehensive figures. Novelty is intermediate to high. There are some points I would like the authors to explain more clearly. Overall, I would recommend a minor revision.

Author response: Thank you for your constructive revisions.

2.1 Main Points

1) The author used a simplified weathering equation to demonstrate: if they vary hydrology parameter, the weathering fluxes would increase as continental fragmentation (CF) proxy increase.

Accordingly, varying temperature parameter in the equation, the weathering fluxes would also increases as TLF proxy increases. This makes sense if we assume the equation is reasonable. However, in later part of the work, the authors then use CF and TLF as proxies for hydrological/temperature controlled weathering (i.e., reverse demonstrating). This does not make sense, because your relationship between CF/TLF proxy and the hydrological/temp controlled-weathering is sufficient but not compulsory. That is to say, changing α or $k \rightarrow$ changing CF/TLF-influenced weathering, but changing CF/TLF-influenced weathering does not solely $\rightarrow$ changing α or k, because other factors may also influencing CF/TLF-derived weathering (e.g., CO_2 level-induced dissolution rate change, tectonics, lithology change, plants colonization, monsoon, etc.), and the authors have roughly acknowledge this point at L237–254). If the paper just said (based on the data), continental configuration influenced global temperature, I would agree.

Author response: You raise a fair point about the logical direction of inference. The GCM results suggest that changing α or k should produce distinct geometric correlations, but it is true that observing those correlations in the geologic record does not uniquely require a shift in α or k, since other factors like lithology, biology, or CO_2-driven kinetics could in principle modulate how weathering responds to continental geometry. However, we think the specific pattern we observe is less likely to be reproduced through those alternative mechanisms. The correlations between fragmentation or TLF and GMST are mutually exclusive and switch at specific times — from TLF-dominated to fragmentation-dominated around 300 Ma and back around 200 Ma. A confounding process would need to influence the weathering–geometry relationship in a way that mimics this switching pattern at times that coincide with Pangea's assembly and breakup. Processes like plant colonization or lithologic change are gradual and largely unidirectional over this interval, making them unlikely candidates for producing two discrete transitions. The coincidence with Pangea is also independently motivated: paleosol and GCM evidence suggest extreme continental aridity during this period, which would push weathering zones toward chemostatic behavior and hydrologic control, consistent with our interpretation.

2) In geological record part, correlation does not mean causality (Fig 4).

Author response: We address various caveats that could weaken our interpretation of the results in the Discussion section. One passage relevant to this point is on Lines 264 to 282:

More generally, given the interconnected and recursive nature of the Earth system, it is difficult to establish causality when analyzing relationships between geological quantities. Although we observe correlations between the variables of interest in this study (i.e., GMST, TLF, and continental fragmentation), it is possible that these relationships are being driven by other mechanisms or variables that are co-varying simultaneously. For example, we excluded variations in CO_2 outgassing rate from our study even though such variations could have played a role in dictating the equilibrium weathering rate and climate state at any given time in Earth history. This is justified given the major uncertainty in reconstructions, with large inconsistencies in the magnitude and direction of outgassing changes depending on the set of processes considered and the methodology used for estimation [Kerrick, 2001, Godderis and Donnadieu, 2019, Coogan and Gillis, 2020, Mills et al., 2021, Marcilly et al., 2021, Müller et al., 2022, Black et al., 2024, Müller et al., 2024, Merdith et al., 2025]. Still, theoretical arguments and some data suggest outgassing may vary systematically across the supercontinent cycle, with

periods of enhanced rifting corresponding to increases in the flux of CO_2 from Earth's interior [e.g., Foley and Fischer, 2017, Brune et al., 2017, Jellinek et al., 2020]. If CO_2 outgassing increases when continental fragmentation increases, then this may systematically bias the system away from displaying an anti-correlation between the degree of fragmentation and global surface temperature, since increased weathering could be partially or completely offset by increased outgassing. If that is the case, then the imprint of hydrologically-controlled weathering on the geologic record might be reduced, biasing our analysis.

3) In geological timescale, CO_2 release (weathering of organic carbon + degassing) = CO_2 removal (mainly silicate weathering) (as authors already suggested). Many studies used a term weathering sensitivity to understand the weathering process in this context (under certain CO_2 level, how much weathering fluxes could be produced). The equation looks like: $W = k \cdot (RCO_2)^a$, where W is weathering flux, k is weatherability, RCO_2 is CO_2 ratio to modern level, and a is a fitting parameter (e.g., see Penmann et al., 2020, ESR). In my eyes, what the authors trying to do in this manuscript is actually to understand what's inside the weatherability term k. If so, personally, I think it would be great if authors could compare the work with previous weatherability works (e.g., Caves Rugenstein et al., 2019 Nature, etc.).

Author response: That's exactly right, weatherability is a good lens to view the results through. We mention this in Lines 127 to 139, to which we have now added some relevant references and noted that weatherability has been suggested as a key driver of climate changes:

We note for clarity that these weathering values are all calculated with the same background CO_2 and global temperature and might therefore be better thought of as bulk "global weatherabilities," since Earth's climate would in reality adjust to bring the global weathering rate into balance with the background CO_2 outgassing rate if a change to continental geometry were to cause a large transient shift in weathering. In other words, these calculations are not intended to imply that the equilibrium global weathering rate has varied by this magnitude over Earth history (since the global rate of equilibrium weathering is set by outgassing), but instead to illustrate the degree to which changes in continental geometry can act as carbon cycle forcings under different assumptions about global weathering controls [Penman et al., 2020]. Several studies have identified changing "weatherability" as a potential major driver behind climate changes during the Phanerozoic Eon due to its influence on the equilibrium point of the carbon cycle and climate system [e.g., Kump and Arthur, 1997, Caves et al., 2016, Caves Rugenstein et al., 2019, Bergmann et al., 2025, Hayes et al., 2025].

2.2 Line-by-Line Comments

This manuscript is well written and thus I only have very little line-by-line comments...

Method: I remembered Josh West 2012 (Geology) used slightly different version of hydrological term in weathering equation. Can you make a comparison how they behave differently?

Author response: Although the West 2012 formulation is somewhat different from the weathering formulations we use in our study, the qualitative relationships between weathering and continental geometry would be similar or identical, since the West 2012 formulation still ultimately calculates weathering as a positive function of runoff and tempera-

ture, with parameters that control the strength of the weathering response to both variables. With parameters resulting in a strong temperature-dependence and weak runoff-dependence, West 2012 weathering should have a strong TLF correlation, and with parameters resulting in strong runoff-dependence and weak temperature-dependence, West 2012 weathering should have strong fragmentation correlation.

L210–214: The timescale in this manuscript is multi-Ma, and I guess it is way longer than 'future lifespan' timescale?

> **Author response:** No, the future lifespan of Earth's biosphere is somewhere between 1 and 2 billion years, depending on how Earth's CO_2 responds to the gradually brightening Sun.

L217–223: Sorry I did not get the logic behind this sentence.

> **Author response:** We added the following to lines 314 to 318 to clarify that we are making the point that the habitable zone concept is fundamentally tied to generalizations of models of Earth's silicate weathering behavior, and so may affect predictions of Earth-like exoplanet climate:
>
> Some even propose to limit the width of the habitable zone based on simulations that suggest that silicate weathering behavior can cause global glaciations for planets receiving somewhat less sunlight than Earth, but the precise details of the weathering parameterization used strongly impact the resulting climate behavior [e.g., Haqq-Misra et al., 2016, Abbot, 2016, Kadoya and Tajika, 2019].

L328: If I understood it right, $W_{\rm ref}$ are different in CF and TLF scenarios. Are the results still comparable?

> **Author response:** Yes, the results are still comparable with different $W_{\rm ref}$. However, to avoid confusion, we slightly revised the weathering formulation to eliminate the need for varying $W_{\rm ref}$ values. The new version normalizes runoff by a reference value $q_{\rm ref}$:
>
> $$W = W_{\rm ref}\left(\frac{q}{q_{\rm ref}}\right)^{\alpha}\exp\big(k(T - T_{\rm ref})\big)$$
>
> The previous formulation, by excluding an explicit $q_{\rm ref}$, effectively assumed $q_{\rm ref} = 1$ m/yr, but realistic global-mean runoff is closer to $\sim$0.25 m/yr. This mismatch meant that at high α, weathering rates were suppressed by roughly a factor of 1/4 relative to what they would be if $q \approx q_{\rm ref}$, while at low α the discrepancy was negligible since runoff has little influence on weathering. Consequently, we had to increase $W_{\rm ref}$ for high α to keep overall weathering rates comparable across cases. By explicitly including $q_{\rm ref} = 0.26$ m/yr—consistent with GCM output—the new formulation allows a single $W_{\rm ref}$ to produce weathering rates of similar magnitude for both high and low α.
>
> Lines 372 to 384 now read:
>
> We take silicate weathering to scale as an exponential function of surface temperature (T) and a power-law function of runoff (q), excluding a parameterization for CO_2 dependence

because all simulations are performed at the same atmospheric CO_2:

$$w \propto \left(\frac{q}{q_{\mathrm{ref}}}\right)^{\alpha} \exp\left(kT\right) \tag{1}$$

$$= w_{\mathrm{ref}} \left(\frac{q}{q_{\mathrm{ref}}}\right)^{\alpha} \exp\big(k(T - T_{\mathrm{ref}})\big) \tag{2}$$

where w [mol m^{-2} yr^{-1}] is the local silicate weathering flux; q [m yr^{-1}] is the local runoff output by the GCM; α is the runoff sensitivity of silicate weathering; T [K] is the local surface temperature output by the GCM; k [K^{-1}] is the temperature sensitivity of weathering; $w_{\mathrm{ref}} = 0.05$ [mol m^{-2} yr^{-1}] is "weatherability" [e.g., Kump and Arthur, 1997] tuned to a value that leads the GCM simulations to produce global weathering rates mostly within the range of ≈3–10 Tmol yr^{-1}, based on estimates of global CO_2 outgassing and analysis of river chemistry [e.g., Gaillardet et al., 1999, Moon et al., 2014, Catling and Kasting, 2017, Coogan and Gillis, 2020]; $q_{\mathrm{ref}} = 0.26$ [m yr^{-1}] is a reference global-mean runoff attained by dividing the estimate for Earth's total modern runoff from Ghiggi et al. [2019] (38452 km^3 yr^{-1}) by Earth's land area ($\approx 148 \times 10^6$ km^2); and $T_{\mathrm{ref}} = 288$ K is a pre-industrial reference temperature [Baum et al., 2022].

The choice of weathering equation: Can I ask why using power law for hydrological control?

Author response: Just a way to vary the parameterization smoothly from no runoff dependence to linear runoff dependence.

Also, can I ask where is the model validation (the Baum et al paper is the validation for your work?)?

Author response: Not sure what you mean. The weathering equation is a slightly modified version of the standard WHAK equation. The global climate model, CESM, is a standard popular choice for simulating Earth climate maintained by NCAR. We didn't really develop any models to validate.

L332: Therefore, terrestrial reverse weathering is not considered?

Author response: It is not clear what you mean by "terrestrial reverse weathering"—reverse weathering usually refers to authigenic clay formation on the seafloor that consumes alkalinity. We do not consider reverse weathering in our calculations.

We added the following to Lines 261 to 263:

Similarly, we do not consider authigenic clay formation ("reverse weathering") which consumes alkalinity and could impact climate equilibria and the response of the carbon cycle to perturbations [e.g., Isson and Planavsky, 2018, Li et al., 2021, Baldermann et al., 2025, Trapp-Müller et al., 2025].

L474: There is a figure in reference?

Author response: That's a Supplemental Information figure that LaTeX placed in the references for some reason.

Finally: I failed to access to the Zenodo link provided. Is it possible to provide a GitHub link?

Author response: Sorry, the Zenodo link should be fixed.

2.3 Reviewer #2 (Remarks on Code Availability)

I am not able to access to the zenodo repository to review the code (i.e., zenodo says the link is broken). I kindly ask whether the authors could provide the github link instead of zenodo (might work better).

> **Author response:** For simplicity we are sticking with zenodo. Sorry about the lack of access previously, we misconfigured permissions originally. The code should now be available at `https://zenodo.org/records/16732631`, and line 463 now has the updated DOI, 10.5281/zenodo.16732631.

3 Reviewer #3 (Remarks to the Author)

Review of "Shifting mechanisms of climate stabilization across the Phanerozoic" (NCOMMS-25-86427) by R.J. Graham et al.

3.1 Summary

Graham et al. analyze the drivers of continental weathering during the Phanerozoic. They first analyze a series of 120 previously-published climatic simulations conducted on idealized continental configurations with a relatively low-resolution version of CESM. They consider temperature-only and runoff-only representations of weathering on land, and demonstrate that total weathering is driven by the low-latitude land area and continental fragmentation, respectively. In a second step, based on these lessons, the authors compare geological reconstructions of global surface air temperature, low-latitude land area and continental fragmentation during the Phanerozoic, in order to identify the periods of time during which continental weathering was driven more by temperature or more by runoff. The authors conclude that temperature was the main driver of continental weathering during the Phanerozoic, with runoff only being the key player during the 300–200 Ma time interval.

3.2 General

I really appreciated the manuscript by Graham et al. It proposes an original approach based on reasonable methods. It is very well written and the figures are of good quality. I support the publication of the manuscript after revisions. Please note that my expertise in weathering is limited; hence I mostly review the general aspect of the manuscript and suggest to contact an expert in weathering for complementary comments.

> **Author response:** Thank you for the thoughtful suggestions.

3.3 Major Points

Paleogeographic sensitivity test. The results largely rely on the geological datasets used. While I acknowledge the large margins of uncertainty associated with climatic reconstructions (see for instance Grossman and Joachimski, 2022), I think that the one of Judd et al. (2024) is a very good choice. Regarding the paleogeographical reconstructions, the authors decided to use the ones of Marcilly et al. (2021). Considering that several alternative Phanerozoic-scale paleogeographical reconstructions are now available in numerical format, I would like to see a sensitivity test that would permit determining the degree of dependence of the conclusions to the dataset used. I suggest to test the reconstructions of the PALEOMAP project (Scotese and Wright, 2018). They are widely used in the deep-time community. Including these additional calculations would be of great interest

to the general readership. These maps are also available every 5 Myrs, which would provide more data points for correlations / statistical tests.

Author response: This is a good idea. We added a figure to the appendix and discussed in the Discussion. Lines 216 to 227 now read:

As a sensitivity test of our results, we repeated the analysis of correlations between surface temperature and exposed continental geometry over the Phanerozoic, replacing the paleogeographic reconstructions from Marcilly et al. [2021] with those of Scotese and Wright [2018]. The two sets of reconstructions differ significantly due to different underlying plate tectonic models [Marcilly et al., 2021], although they are not entirely independent, since Marcilly et al. [2021] relied on some of the same data sources as Scotese and Wright [2018]. With the Scotese reconstruction (see Figure A2), our results are qualitatively similar, with a significantly negative relationship between TLF and GMST from 480 Ma to 300 Ma ($r = -0.70$, $p = 2.69 \times 10^{-4}\%$) and a significantly negative relationship between fragmentation and GMST from 300 Ma to 200 Ma ($r = -0.62$, $p = 0.214\%$), but the anti-correlation between TLF and GMST from 200 Ma to present is weaker ($r = -0.31$) and marginally significant ($p = 5.39\%$).

Volcanic outgassing assumption. Lines 151–164: this rationale supposes an invariant volcanic outgassing rate, i.e., that global temperature is controlled only by the CO_2 sink with no substantial contribution of the source. This strong assumption is discussed on lines 263–268; considering its first-order impact, I suggest to state that up front. In particular, fragmentation and volcanism are probably closely related, with periods of continental fragmentation (hence mid-ocean ridge activity) probably associated with strong volcanic outgassing rates? If so, a strong fragmentation would be associated with high volcanic outgassing rates hence presumably warmer climates in the paleoclimate reconstructions. I think the results are thus biased towards an underestimation of the impact of the hydrological cycle, which may explain why only 20% of the time interval is found to be associated with a runoff-dominated weathering regime? I think this point deserves some in-depth discussion.

Author response: We have altered the paper to bring up the constant outgassing earlier. Lines 96 to 102 now read:

Ultimately, our calculations are consistent with the hypothesis that Earth's weathering control mechanism shifted twice in the last 480 Ma, from a temperature-based to a hydrology-based configuration ≈300 Ma and then back to a temperature-based configuration ≈200 Ma, though this is dataset-dependent and relies on assumptions like weakly varying outgassing. Still, the results provide useful new insight into the structure of the carbon cycle which may lead to improved understanding of the evolution of Earth's climate over the Phanerozoic.

We also expanded our discussion of outgassing slightly to include your point about the possible covariation between fragmentation and outgassing. Lines 269 to 282 now read:

For example, we excluded variations in CO_2 outgassing rate from our study even though such variations could have played a role in dictating the equilibrium weathering rate and climate state at any given time in Earth history. This is justified given the major uncertainty in reconstructions, with large inconsistencies in the magnitude and direction of outgassing changes depending on the set of processes considered and the methodology used for estimation [Kerrick, 2001, Godderis and Donnadieu, 2019, Coogan and Gillis, 2020, Mills et al., 2021, Marcilly et al., 2021, Müller et al., 2022, Black et al.,

2024, Müller et al., 2024, Merdith et al., 2025]. Still, theoretical arguments and some data suggest outgassing may vary systematically across the supercontinent cycle, with periods of enhanced rifting corresponding to increases in the flux of CO_2 from Earth's interior [e.g., Foley and Fischer, 2017, Brune et al., 2017, Jellinek et al., 2020]. If CO_2 outgassing increases when continental fragmentation increases, then this may systematically bias the system away from displaying an anti-correlation between the degree of fragmentation and global surface temperature, since increased weathering could be partially or completely offset by increased outgassing. If that is the case, then the imprint of hydrologically-controlled weathering on the geologic record might be reduced, biasing our analysis.

3.4 Secondary Points

Accessibility of methods. I had to read the Methods and have a look at Baum et al. (2022) at the start of section "Sensitivity of silicate weathering to tropical land fraction…". I think it is important to include a short summary of the key elements here to make the manuscript accessible to a reader with no previous knowledge of Baum et al. (2022). In particular, the fact that the 120 continental configurations are idealized was at first a bit surprising for me; I suggest to introduce such key notions here, as well as key notions regarding the calculations of weathering and the fact that simulations were conducted using a constant pCO_2, etc. Actually, it may help to state as early as in the abstract, that the continental configurations are idealized.

Author response: The Abstract now contains the sentence:

Here, we investigate this fundamental question by analyzing a large ensemble of Community Earth System Model (CESM) global climate model (GCM) simulations with different idealized, randomized continental configurations, which we post-process to calculate silicate weathering with systematically varying temperature- and runoff-sensitivities.

And Lines 88 to 92 now read:

Here, to assist in differentiating between signatures of hydrology-controlled and temperature-controlled weathering in Earth history, we post-process the runoff and temperature fields to estimate global weathering rates from a large ensemble of GCM simulations that are identical except for their randomized continental configurations.

Lines 123–125: The authors state that "hydrological controlled weathering […] is not strongly affected by TLF". I don't think this is exact; TLF can induce a doubling of weathering (from 1.8 to 4.0), close to the impact of fragmentation. I think the key point here is that weathering does not change monotonically with TLF.

Author response: We have added a new figure (figure 4) showing how runoff, global-mean surface temperature, and global-mean land temperature all vary with TLF and continental fragmentation. We altered lines 152 to 171 to read:

The reason for this divergence in response to continental geometry is straightforward. Within our GCM ensemble, global runoff is uncorrelated with TLF ($R^2 = 0\%$) but strongly correlated with continental fragmentation ($R^2 = 54\%$; see Fig. 4a, b). Therefore, when weathering is dominated by hydrology, changes to TLF have a minimal effect, but changes to fragmentation can alter weathering rates through their impact on runoff, with more fragmented configurations experiencing active rainfall and runoff over a larger

fraction of their land. Conversely, although global-mean surface temperature is both barely correlated with TLF ($R^2 = 16\%$) and uncorrelated with continental fragmentation ($R^2 = 0\%$; see Fig. 4c, d), global-mean *land* temperature is strongly correlated with TLF ($R^2 = 71\%$), while remaining uncorrelated with fragmentation ($R^2 = 1\%$; see Fig. 4e, f). So, when temperature is the main driver of weathering, changes to TLF can change the weathering rate by altering the amount of land that experiences higher temperatures near the equator. The small positive relationship between global-mean surface temperature and TLF visible in Fig. 4c is due to a combination of the reduction of high-albedo clouds when tropical ocean is replaced with mostly cloud-free land in high-TLF simulations, as well as an increase in high-albedo land snow in low-TLF simulations where land is colder and closer to the poles. Inclusion of ocean heat transport could modulate this effect by redistributing energy from the tropics toward the poles, reducing or eliminating the correlation entirely [Jenkins, 1995].

Reverse weathering. I think that the authors may also want to discuss the potential modulation of weathering by reverse weathering in their discussion (e.g., Isson and Rauzi, 2024).

Author response: Lines 261 to 263 now read:

Similarly, we do not consider authigenic clay formation ("reverse weathering") which consumes alkalinity and could impact climate equilibria and the response of the carbon cycle to perturbations [e.g., Isson and Planavsky, 2018, Li et al., 2021, Baldermann et al., 2025, Trapp-Müller et al., 2025].

Fig. 4 time interval. From Fig. 4, it seems that the interval 485 to ∼420 Ma may rather be hydrology-controlled? At least, weathering at that time does not seem to be temperature-controlled, based on the apparent co-variation of GMST and TLF, after the top and bottom-right panels. Excluding these time slices from the correlation may increase the p-value (of 0.025 after line 172?). Similarly, I guess one could discuss the controls of weathering over the last ∼25 Myrs after Fig. 4.

Author response: You may be right, and with more granular datasets those points are worth looking into more closely, but we hesitate to pick and choose the datapoints like this for fear of over-interpretation. But the point is well-taken.

3.5 Technical Comments

- Line 32: a period is missing.
- Lines 83–88: please consider splitting the very long sentence.

  Author response: Lines 88 to 94 now read:

  Here, to assist in differentiating between signatures of hydrology-controlled and temperature-controlled weathering in Earth history, we post-process the runoff and temperature fields to estimate global weathering rates from a large ensemble of GCM simulations that are identical except for their randomized continental configurations. This allows us to demonstrate that these alternative weathering controls lead to alternative predictions for Earth's climate response to changes in aspects of bulk planetary continental geometry.
- Lines 245–248: the authors may consider citing also the mechanistic modeling approach of Maffre et al. (2022).

Author response: Line 254 now contains a reference to Maffre et al. (2022).

- Caption of Fig. 1: the description of high/low fragmentation index is reversed.

  Author response: Fixed.

- Caption of Fig. 4: missing space in first line.

  Author response: Fixed.

- Fig. 4: need to add some space on the right of "tropical land fraction" to avoid overlapping with the 3rd y-axis. + I suggest to add *p*-values to bottom panels.

  Author response: Done.

- Please remind the reader where GCM fields can be publicly accessed for convenience.

  Author response: Lines 343 to 348 now read:

  We use an ensemble of Community Earth System Model (CESM) global climate model (GCM) simulations originally presented by Baum et al. [2022] and available for download at Baum and Fu [2022] to analyze the sensitivity of silicate weathering rates to tropical land fraction (TLF) and continental fragmentation, represented by the ratio of non-submerged continental perimeter to continental area, which we denote the "continental fragmentation index" following Gernon et al. [2021].

  And the data availability statement now includes:

  The GCM simulations are available at Baum and Fu [2022].

3.6 Reviewer #3 (Remarks on Code Availability)

Permission required: "You do not have sufficient permissions to view this page."

Author response: Sorry, we misconfigured the permissions. The code should now be available at `https://zenodo.org/records/16732631`, and line 463 now has the updated DOI, 10.5281/zenodo.16732631.

3.7 References Cited by Reviewer 3

- Baum, M., Fu, M., Bourguet, S., 2022. Sensitive Dependence of Global Climate to Continental Geometry. *Geophysical Research Letters* 49, 1–8.
- Goddéris, Y., Donnadieu, Y., Carretier, S., Aretz, M., Dera, G., MacOuin, M., Regard, V., 2017. Onset and ending of the late Palaeozoic ice age triggered by tectonically paced rock weathering. *Nature Geoscience* 10, 382–386.
- Grossman, E.L., Joachimski, M.M., 2022. Ocean temperatures through the Phanerozoic reassessed. *Scientific Reports*, 1–14.
- Isson, T., Rauzi, S., 2024. Oxygen isotope ensemble reveals Earth's seawater, temperature, and carbon cycle history. *Science* 383, 666–670.
- Judd, E.J., Tierney, J.E., Lunt, D.J., Montañez, I.P., Huber, B.T., Wing, S.L., Valdes, P.J., 2024. A 485-million-year history of Earth's surface temperature. *Science* 385, eadk3705.

- Maffre, P., Chiang, J.C.H., Swanson-Hysell, N.L., 2023. The effect of the Pliocene temperature pattern on silicate weathering and Pliocene–Pleistocene cooling. *Clim. Past* 19, 1461–1479.
- Maffre, P., Goddéris, Y., Le Hir, G., Nardin, É., Sarr, A.-C., Donnadieu, Y., 2025. GEOCLIM7, an Earth system model for multi-million-year evolution of the geochemical cycles and climate. *Geosci. Model Dev.* 18, 6367–6413.
- Maffre, P., Goddéris, Y., Pohl, A., Donnadieu, Y., Carretier, S., Le Hir, G., 2022. The complex response of continental silicate rock weathering to the colonization of the continents by vascular plants in the Devonian. *American Journal of Science* 322, 461–492.
- Marcilly, C.M., Torsvik, T.H., Domeier, M., Royer, D.L., 2021. New paleogeographic and degassing parameters for long-term carbon cycle models. *Gondwana Research*.
- Martin, P.E., Macdonald, F.A., McQuarrie, N., Flowers, R.M., Maffre, P.J.Y., 2023. The rise of New Guinea and the fall of Neogene global temperatures. *Proc. Natl. Acad. Sci. U.S.A.* 120, e2306492120.

References

D. S. Abbot. Analytical investigation of the decrease in the size of the habitable zone due to limited co_2 outgassing rate. *The Astrophysical Journal*, 827:117, 2016.

Andre Baldermann, Santanu Banerjee, Stefan C Löhr, Maxim Rudmin, Laurence N Warr, and Arpita Chakraborty. Exploring reverse silicate weathering across geological time: a review. *Clay Minerals*, 60(1):1–27, 2025.

Mark Baum and Minmin Fu. Ensemble weathering results & project files, February 2022. URL https://doi.org/10.5281/zenodo.6540321.

Mark Baum, Minmin Fu, and Stephen Bourguet. Sensitive dependence of global climate to continental geometry. *Geophysical Research Letters*, page e2022GL098843, 2022.

Kristin D Bergmann, Francis A Macdonald, and Nicholas L Swanson-Hysell. The causes and consequences of ordovician cooling. *Annual Review of Earth and Planetary Sciences*, 53, 2025.

Benjamin A Black, Leif Karlstrom, Benjamin JW Mills, Tamsin A Mather, Maxwell L Rudolph, Jack Longman, and Andrew Merdith. Cryptic degassing and protracted greenhouse climates after flood basalt events. *Nature Geoscience*, 17(11):1162–1168, 2024.

Wallace S Broecker and Abhijit Sanyal. Does atmospheric co2 police the rate of chemical weathering? *Global Biogeochemical Cycles*, 12(3):403–408, 1998.

Sascha Brune, Simon E Williams, and R Dietmar Müller. Potential links between continental rifting, co2 degassing and climate change through time. *Nature Geoscience*, 10(12):941–946, 2017.

David C Catling and James F Kasting. *Atmospheric evolution on inhabited and lifeless worlds*. Cambridge University Press, 2017.

Jeremy K Caves, Adam B Jost, Kimberly V Lau, and Kate Maher. Cenozoic carbon cycle imbalances and a variable weathering feedback. *Earth and Planetary Science Letters*, 450:152–163, 2016.

Jeremy K Caves Rugenstein, Daniel E Ibarra, and Friedhelm von Blanckenburg. Neogene cooling driven by land surface reactivity rather than increased weathering fluxes. *Nature*, 571(7763): 99–102, 2019.

LA Coogan and KM Gillis. The average phanerozoic co2 degassing flux estimated from the o-isotopic composition of seawater. *Earth and Planetary Science Letters*, 536:116151, 2020.

Stephen F Foley and Tobias P Fischer. An essential role for continental rifts and lithosphere in the deep carbon cycle. *Nature Geoscience*, 10(12):897–902, 2017.

J Gaillardet, B Dupré, Pl Louvat, and CJ Allegre. Global silicate weathering and co2 consumption rates deduced from the chemistry of large rivers. *Chemical geology*, 159(1-4):3–30, 1999.

Thomas M Gernon, Thea K Hincks, Andrew S Merdith, Eelco J Rohling, Martin R Palmer, Gavin L Foster, Clément P Bataille, and R Dietmar Müller. Global chemical weathering dominated by continental arcs since the mid-palaeozoic. *Nature Geoscience*, 14(9):690–696, 2021.

Gionata Ghiggi, Vincent Humphrey, Sonia I Seneviratne, and Lukas Gudmundsson. Grun: an observation-based global gridded runoff dataset from 1902 to 2014. *Earth System Science Data*, 11(4):1655–1674, 2019.

Yves Godderis and Yannick Donnadieu. A sink-or a source-driven carbon cycle at the geological timescale? relative importance of palaeogeography versus solid earth degassing rate in the phanerozoic climatic evolution. *Geological Magazine*, 156(2):355–365, 2019.

Jacob Haqq-Misra, Ravi Kumar Kopparapu, Natasha E Batalha, Chester E Harman, and James F Kasting. Limit cycles can reduce the width of the habitable zone. *The Astrophysical Journal*, 827 (2):120, 2016.

Nick R Hayes, Daniel J Lunt, Yves Goddéris, Richard D Pancost, and Heather Buss. Modelling the impact of palaeogeographical changes on weathering and co2 during the cretaceous–eocene period. *Climate of the Past*, 21(12):2601–2618, 2025.

Dominik Hülse and Andy Ridgwell. Instability in the geological regulation of earth's climate. *Science*, 389(6767):eadh7730, 2025.

Terry T Isson and Noah J Planavsky. Reverse weathering as a long-term stabilizer of marine ph and planetary climate. *Nature*, 560(7719):471, 2018.

AM Jellinek, A Lenardic, and RT Pierrehumbert. Ice, fire, or fizzle: The climate footprint of earth's supercontinental cycles. *Geochemistry, Geophysics, Geosystems*, 21(2):e2019GC008464, 2020.

Gregory S Jenkins. Early earth's climate: Cloud feedback from reduced land fraction and ozone concentrations. *Geophysical research letters*, 22(12):1513–1516, 1995.

Emily J Judd, Jessica E Tierney, Daniel J Lunt, Isabel P Montañez, Brian T Huber, Scott L Wing, and Paul J Valdes. A 485-million-year history of earth's surface temperature. *science*, 385(6715): eadk3705, 2024.

S Kadoya and E Tajika. Outer limits of the habitable zones in terms of climate mode and climate evolution of earth-like planets. *The Astrophysical Journal*, 875(1):7, 2019.

Derrill M Kerrick. Present and past nonanthropogenic co2 degassing from the solid earth. *Reviews of Geophysics*, 39(4):565–585, 2001.

Lee R Kump. Prolonged late permian–early triassic hyperthermal: failure of climate regulation? *Philosophical Transactions of the Royal Society A: Mathematical, Physical and Engineering Sciences*, 376(2130):20170078, 2018.

Lee R Kump and Michael A Arthur. Global chemical erosion during the cenozoic: Weatherability balances the budgets. In *Tectonic uplift and climate change*, pages 399–426. Springer, 1997.

Fangbing Li, Donald Penman, Noah Planavsky, Andrew Knudsen, Mingyu Zhao, Xiangli Wang, Terry Isson, Kangjun Huang, Guangyi Wei, Shuang Zhang, et al. Reverse weathering may amplify post-snowball atmospheric carbon dioxide levels. *Precambrian Research*, 364:106279, 2021.

Chloé M Marcilly, Trond H Torsvik, Mathew Domeier, and Dana L Royer. New paleogeographic and degassing parameters for long-term carbon cycle models. *Gondwana Research*, 97:176–203, 2021.

Andrew S Merdith, Thomas M Gernon, Pierre Maffre, Yannick Donnadieu, Yves Goddéris, Jack Longman, R Dietmar Müller, and Benjamin JW Mills. Phanerozoic icehouse climates as the result of multiple solid-earth cooling mechanisms. *Science Advances*, 11(7):eadm9798, 2025.

Benjamin JW Mills, Yannick Donnadieu, and Yves Goddéris. Spatial continuous integration of phanerozoic global biogeochemistry and climate. *Gondwana Research*, 2021.

Seulgi Moon, CP Chamberlain, and GE Hilley. New estimates of silicate weathering rates and their uncertainties in global rivers. *Geochimica et Cosmochimica Acta*, 134:257–274, 2014.

R Dietmar Müller, Ben Mather, Adriana Dutkiewicz, Tobias Keller, Andrew Merdith, Christopher M Gonzalez, Weronika Gorczyk, and Sabin Zahirovic. Evolution of earth's tectonic carbon conveyor belt. *Nature*, 605(7911):629–639, 2022.

R Dietmar Müller, Adriana Dutkiewicz, Sabin Zahirovic, Andrew S Merdith, Christopher R Scotese, Benjamin JW Mills, Lauren Ilano, and Ben Mather. Solid earth carbon degassing and sequestration since 1 billion years ago. *Geochemistry, Geophysics, Geosystems*, 25(11):e2024GC011713, 2024.

Donald E Penman, Jeremy K Caves Rugenstein, Daniel E Ibarra, and Matthew J Winnick. Silicate weathering as a feedback and forcing in earth's climate and carbon cycle. *Earth-Science Reviews*, page 103298, 2020.

Christopher R Scotese and Nicky Wright. Paleomap paleodigital elevation models (paleodems) for the phanerozoic. *Paleomap Proj*, 2018.

Gerrit Trapp-Müller, Jeremy Caves Rugenstein, Daniel J Conley, Sonja Geilert, Mathilde Hagens, Wei-Li Hong, Catherine Jeandel, Jack Longman, Paul RD Mason, Jack J Middelburg, et al. Earth's silicate weathering continuum. *Nature Geoscience*, 18(8):691–701, 2025.

Richard E Zeebe and Ken Caldeira. Close mass balance of long-term carbon fluxes from ice-core co 2 and ocean chemistry records. *Nature Geoscience*, 1(5):312, 2008.

Reviewer Comments and Author Responses

"Shifting mechanisms of climate stabilization across the Phanerozoic" (now "Paleogeographic controls on Phanerozoic weathering rates and implications for climate regulation") by Graham et al.

Contents

1 Reviewer #1 (Remarks to the Author) 2

1.1 General Comments 2

1.2 New minor comments: 6

2 Reviewer #2 (Remarks to the Author) 7

2.1 Few points: 7

1 Reviewer #1 (Remarks to the Author)

I thank the authors for addressing the comments raised by myself and other reviewers. Please find my thoughts on each comment below in green. I am generally content with the changes the authors have made but, in some instances, the original comments could/should be addressed with more consideration. I particularly like the new Figure 4 and the new discussion on the sensitivity of the result to choice of paleogeographic reconstruction–both improved the manuscript.

This remains an incredibly interesting study, using an original approach to study what physical processes contributed to the large-scale evolution of Earth's climate across time. I highly recommend publication; resubmit with minor corrections.

Regards, Pam Vervoort

> **Author response:** Thank you for the encouraging words and the useful critiques. We have responded by
>
> - expanding the paper's discussion of previous studies examining interactions between paleogeography, climate, and weathering
> - explaining in the paper why runoff does not strongly depend on tropical land fraction
> - explaining the weak relationship between TLF and global-mean surface temperature
> - adding mention of the smaller ensembles of 500 ppm and 2000 ppm simulations that showed consistent results with our larger 1000 ppm ensemble
> - making small adjustments to wording according to your suggestions
>
> We appreciate your time and effort.

1.1 General Comments

The first part of the manuscript demonstrates clearly that either fragmentation or TLF is important, depending on the dominant weathering rates control. These results are really interesting, and it would benefit from a more extended investigation into why this is. On Lines 138–150 reasons are given for why temperature-dependent weathering is mostly linked to TLF while runoff-dependent weathering is mostly linked to fragmentation. While straightforward, the reader needs to see the evidence to back this up. In current form, the reader cannot judge how temperature and runoff change with fragmentation and TLF. For example, I imagine that temperature and fragmentation are not entirely decoupled as fragmentation affects ocean-atmosphere interaction. Especially the statement "[...] changing TLF does not tend to lead to large changes in runoff, [...]" needs further explanation; this is not per se intuitive. An increase in TLF can also increase runoff as more land is located in high-precipitation regions.

Old author response: We have added a new figure (figure 4) showing how runoff, global-mean surface temperature, and global-mean land temperature all vary with TLF and continental fragmentation. We altered lines 152 to 171 to read:...

Reviewer response: New Fig 4 is a nice addition that clarifies the conclusions drawn. It is clear why there is a relation between TLF and land surface temp, however, I'm still unsure why TLF does not influence runoff. I can see R2=0 in panel a but I'm looking for a physical explanation as

opposed to simply showing 'no correlation'. Would an increase in TLF not lead to more runoff with more land in high-precipitation regions? I'm actually not sure about the answer but substantiating this model behaviour with 'real world' or modelled physical mechanisms would help me and other readers understand. This point is important because the result of the papers rely on this. (For context: I'm thinking about the Amazon River. Amazon land lies in the tropical region and it's one of the rivers with the largest runoff rates today).

Author response: It is true that precipitation over land increases as TLF increases: the partial correlation between precipitation averaged over land and TLF (controlling for fragmentation) is $r = 0.57$ with a $p-$value of 9.74×10^{-12}. However, runoff is the difference between precipitation and evaporation over land. Evaporation is constrained by moisture supply (precipitation over land) and energy supply (difference between absorbed solar radiation and net longwave). As TLF increases, the land on average intercepts more radiation, which means the surface can re-evaporate a larger fraction of the rain that falls onto it. So, both the magnitude of land precipitation and the fraction of land precipitation that gets evaporated increase with TLF, and these effects cancel, leading to a roughly constant difference between precipitation and evaporation, meaning a roughly constant global rate of runoff. Multiple linear regression of global runoff against global land precipitation and global-mean net surface radiation confirms this picture with $R^2 = 0.904$ and normalized slopes of $\beta_{\text{precip}} = 1.04$ and $\beta_{\text{rad}} = -0.407$, both with small variance inflation factors of 1.21 implying minor correlation between the predictors. This shows that the variation in global runoff is almost entirely explained by variations in supply of moisture (which increases runoff by increasing precipitation) and energy (which decreases runoff by increasing evaporation) to the continents. Consistent with this, regressing evaporation from land against the same two variables (land precipitation and net land radiation) results in $R^2 = 0.958$ with $\beta_{\text{precip}} = 0.763$ and $\beta_{\text{rad}} = 0.374$ and the same 1.21 variance inflation factors as before. Another confirmation of this point: If we control for land precipitation, then the correlation between runoff and TLF is strongly negative due to the evaporation effect, with $r = -0.801$ and $p = 7.5 \times 10^{-28}$. In other words, for two simulations with identical total land precipitation but different TLF, the simulation with higher TLF will tend to have lower runoff due to amplified land evaporation.

To the point about the Amazon: Regardless of TLF, runoff peaks in the tropics in all cases, consistent with the presence of the Amazon on the modern Earth. Intuitively, increasing TLF places more land in the wettest region of the planet, but it also progressively decreases the atmosphere's main moisture source by replacing tropical ocean with land. This leads to a negative correlation between TLF and total global precipitation (land + ocean), and contributes to a disproportionate decrease in moisture supply to continents in the extratropics, meaning runoff in the extratropics decreases more than would be predicted just based on the decrease in extratropical land area with increasing TLF (i.e., rainfall per unit land area in the extratropics decreases along with the actual decrease in extratropical land). In fact, the precipitation and runoff per unit land area decreases across all latitudes as TLF increases, but the increased land area in the tropics with increasing TLF is large enough that overall tropical runoff still rises. This overall increase in tropical runoff is offset by the extratropical runoff decrease.

To clarify this, lines 174 to 183 now read:

The lack of correlation between TLF and runoff is due to an increase in land evaporation

in high-TLF simulations that compensates for increased land precipitation, similar to an effect observed by Otto-Bliesner [1995]. This occurs because tropical land intercepts more solar energy, allowing for more efficient recycling of water despite increased overall delivery to the land via precipitation in the wet tropics. Increasing fragmentation does not increase delivery of solar energy to land, but it does increase delivery of water, so runoff tends to increase as continents break up. So, when weathering is dominated by hydrology, changes to TLF have a minimal effect due to small changes in runoff, but more fragmented configurations produce more runoff, accelerating weathering.

1. The authors assume a direct link between CO_2 and temperature which is perhaps somewhat simplistic. While it is true for most of the Phanerozoic, Judd et al. (2024) also point out that there is no strong correlation between CO_2 and GMST during the Mesozoic (250–66 Ma). Under the same CO_2, global mean temperatures can differ when the land mass configuration is different. Lands have a different heat capacity and albedo profile than oceans, and changes in heat distribution by ocean currents change with landmass. In addition, some paleogeographies are more prone to develop ice sheets with can lead to further decoupling of GMST and CO_2. This point needs to be addressed in my opinion to back up why GMST can be used to infer weathering regimes. A major benefit of the idealized GCM ensemble is that you could identify the extent to which landmass distribution affects the global temperature (under fixed CO_2) and use that to discuss the implications for the Phanerozoic GMST–CO_2 relationships.

Old author response: In addition to the addition of Figure 4 and discussion of the relationships between temperature, TLF, and fragmentation noted above, we have now added the following to lines 235 to 242...

Reviewer response: Thanks for clarifying. I appreciate acknowledgement of the uncertainties surrounding the GMST-CO2 relation. Stating the positive correlation between GMST and CO2 is a first-order assumption is necessary. The new Fig 4cd suggest that paleogeography has minimal influence on the GMST though panel c shows that there is still a positive R2=16% relation between TLF and GMST. Is this statistically significant? I am still of the opinion that a more in-depth discussion or analysis of how paleogeography, especially latitudinal landmass distribution, affects GMST (all else being equal) is appropriate. E.g. discuss existing and very relevant literature (e.g. Worsley et al., 1994 or Lunt et al., 2016, and others)?

Author response: Yes, the 16% R^2 between TLF and global-mean temperature is statistically significant ($p = 5 \times 10^{-6}$). Global-mean temperature variation in our simulations is entirely a function of variation in albedo (global-mean temperature and planetary albedo have a correlation coefficient of $r = -0.994$). The small increase in global-mean temperature with increasing TLF is an effect of exchanging snow on land at high latitudes for sea ice as land migrates into the tropics. In these simulations, sea ice has a lower albedo than snow on land, so when the high latitudes are predominantly ocean instead of land, the planetary albedo is slightly lower.

We have clarified this point in Lines to 190 to 198:

The small positive relationship between global-mean surface temperature and TLF visible in Fig. 4c is due to an increase in high-albedo land snow in low-TLF simulations where land is colder and closer to the poles, since snow on land has higher albedo than sea ice in these simulations. Warmer global conditions moderate this effect: a smaller ensemble of simulations with 30 of the 120 paleogeographies from this paper and Baum et al. [2022] with doubled CO_2 (2000 ppm; not shown) showed a TLF-global tempera-

ture correlation with an even weaker R^2 of 2% due to the reduced latitudinal extent of freezing temperatures while preserving a strong TLF-land temperature correlation.

——

We have also expanded the discussion of relationships between continental configuration and climate. In the discussion, we added the Lunt reference (which finds a small direct impact of paleogeography with unclear mechanism).

Lines 269 to 273 now read:

This increases our confidence that it is a reasonable first-order assumption that CO_2 and temperature were tightly linked over most of the Phanerozoic and that variations in continental geometry were unlikely to have a large direct (non-carbon-cycle-mediated) effect on surface temperatures, consistent with previous GCM ensemble studies [e.g., Otto-Bliesner, 1995, Lunt et al., 2016].

——

The Worsley papers and other similar works attempt to quantify the weathering effect of paleogeography based on various assumed weathering parameterizations, which is of course extremely relevant to our paper, so we added a paragraph to the introduction touching on some of this work.

Lines 88 to 107 now read:

Although the behavior of silicate weathering and its interaction with the climate is still subject to debate, a substantial volume of work attempts to connect changes in paleogeography during the Phanerozoic Eon to changes in climate and CO_2 through the weathering feedback using models of widely varying complexity [e.g., Worsley and Kidder, 1991, Worsley et al., 1994, Donnadieu et al., 2004, Berner, 2006, Donnadieu et al., 2006a,b, Goddéris et al., 2014, Godderis and Donnadieu, 2019, Mills et al., 2021, Kemeny et al., 2025]. Early models like Worsley and Kidder [1991], Worsley et al. [1994] assumed a strong temperature control on weathering with no direct runoff-dependence and used bulk estimates of land area and average landmass latitude to predict climate as a function of paleogeography. The GEOCARB and GEOCARBSULF lineage of global-mean box models [Berner, 2006] used relationships derived from GCM studies like that of Otto-Bliesner [1995] to represent the impact of paleogeography on global-mean runoff and temperature, allowing for an effect on climate through a global weathering relationship based on both an assumed strong temperature sensitivity and a moderate runoff sensitivity. The GEOCLIM lineage of models [e.g., Goddéris et al., 2014, Godderis and Donnadieu, 2019] and successors like SCION [Mills et al., 2021] couple weathering parameterizations with strong temperature-dependence and strong (linear) runoff-dependence to output from GCM simulations in order to calculate spatially resolved weathering fluxes, allowing for investigations of climate and CO_2 evolution that directly account for paleogeographic changes in Earth history [e.g., Donnadieu et al., 2006b,a, Godderis et al., 2007, Nardin et al., 2011, Park et al., 2020, Gurung et al., 2024, Longman et al., 2025, Zhang et al., 2026]. However, these models all take their chosen weathering feedback strengths for temperature and runoff as given (modulo occasional sensitivity tests), introducing a major source of uncertainty into their results and reconstructions.

Further, I am wondering about the role of background climate state. The Phanerozoic has seen

large climate change. Paleoclimate proxy records suggest that at higher global temperatures, the latitudinal temperature gradient is less steep and hydrological cycling is more intense, presumably with consequences for the importance of TLF and fragmentation in controlling the weathering strengths?

Old author response: Latitudinal temperature gradient has varied by a bit less than a factor of two over the past 95 million years, which encompasses the hottest period of the Phanerozoic, corresponding to a minimum equator-to-pole temperature gradient of 15.6 C (Gaskell et al., 2022). If we assume a fairly weak temperature feedback with an e-folding temperature of 30 C (corresponding to k = 1/30 C-1), then teleporting a continent from pole to equator would multiply weathering by a factor of $\exp \frac{15.6}{30} \approx 1.7$. With a stronger e-folding temperature of 15 C (k=1/15 C-1), movement from pole to equator would increase weathering by a factor $\exp \frac{15.6}{15} \approx 5.9$. In other words, with the weakest expected equator-to-pole temperature gradient, TLF should still significantly impact weathering rates even if the temperature feedback is weak, and if the temperature feedback is strong, the effect can still be quite large.

Regarding changes to hydrological cycling, if weathering is primarily temperature-dependent, then more intense hydrological cycling won't have much impact on weathering. If weathering is primarily hydrologically-mediated, then more intense hydrological cycling will lead to increased weathering, since that is the basic feedback mechanism. Unless the background temperature state is somehow influencing the feedback mechanism, it shouldn't affect the point that fragmentation is important for hydrologically-controlled weathering but not for temperature-controlled weathering. In a set of calculations not included in the manuscript, we looked into this a bit using a subset of the idealized paleogeographies in the paper (just 10 instead of 40), evaluating the correlations at 500 ppm and 2000 ppm (vs. 1000 ppm in the paper); the same bulk correlations appear.

Reviewer response: Indeed, I was mainly wondering if the background climate state influences the feedback mechanism because a different meridional temperature gradient affects hydrology and temperature patterns. It's important information that the results at 500 ppm and 2000 ppm are comparable to those you find at 1000 ppm! In fact, that would be useful to mention in the manuscript

> **Author response:** True.
>
> Lines 193 to 201 now read:
>
> Warmer global conditions moderate this effect: a smaller ensemble of simulations with 30 of the 120 paleogeographies from this paper and Baum et al. [2022] with doubled CO_2 (2000 ppm; not shown) showed a TLF-global temperature correlation with an even weaker R^2 of 2% due to the reduced latitudinal extent of freezing temperatures while preserving a strong TLF-land temperature correlation. In general, the 30 warmer simulations showed the same basic behavior and relationships as the simulations seen in Fig. 4, as did a set of 30 colder simulations (500 ppm CO_2; not shown) with the same subset of paleogeographies as the warmer simulations.

1.2 New minor comments:

L. 100: "though this relies on assumptions like weakly varying outgassing." And the assumed simplified relation between CO2 and global mean temperature? And, especially further back in time, uncertainties in paleogeographic reconstructions?

> **Author response:** Lines 120 to 122 now read:

...though this relies on assumptions like weakly varying outgassing, a tight relationship between CO_2 and surface temperature, and the accuracy of uncertain paleogeographic reconstructions.

L. 172: "These distinctive differences in the response of the carbon cycle to continental geometry naturally lead to a hypothesis:" Do you mean '. . . response of the weathering feedback to continental geometry. . . .'?

Author response: Either one works, but the weathering feedback is the component of the carbon cycle we focus on in this manuscript, so lines 202 to 203 now read:

These distinctive differences in the response of the weathering feedback to continental geometry naturally lead to a hypothesis...

2 Reviewer #2 (Remarks to the Author)

It great to see the authors have made a detailed reply and according changes in the revised manuscript. Major points (from the 1st round comment) have been resolved and discussed with details (L243-263, L264-282). Influences of choosing the power-law style equation to overall results have been discussed. The code is also available right now. From my side, this manuscript is well-written.

Author response: Excellent, we are happy we successfully addressed most of your concerns and we appreciate the kind words. We have responded to your remaining points below.

2.1 Few points:

1. Remind readers, GMST is a proxy for CO2 and ultimately the proxy for weathering flux at each timestep.

Author response: It's not exactly true to say that CO_2 is a proxy for weathering flux: weathering rate on long timescales is equal to degassing rate of CO_2 from Earth's interior. For the purposes of our calculations, we assume degassing remained constant during the Phanerozoic Eon, which would translate to a constant weathering at steady-state. The CO_2 would be better thought of as a proxy for global "weatherability"– high CO_2 implies the land surface is not weathering efficiently per unit area, so CO_2 has accumulated to produce a large enough weathering flux to balance CO_2 degassing, and low CO_2 means the land surface is weathering extra efficiently, so CO_2 had to be drawn down to a level low enough to balance with outgassing. Assuming constant degassing, any global changes to weathering flux are transient and act to re-equilibrate the global weathering rate with the degassing rate. For example, if we assume weathering is temperature-dependent and the planet's weathering rate (W) is in equilibrium with a CO_2 outgassing rate V, and then we impose an increase in TLF, the weathering rate would initially increase ($W > V$), but this would mean degassing would now be delivering CO_2 to the atmosphere more slowly than its removal by weathering, so CO_2 would fall until weathering slowed down to equal the rate of CO_2 degassing. The net final effect of an increase in TLF in this scenario would then be a colder climate with the same weathering rate as before, after a (geologically short, 10^5 to 10^6 year) period of adjustment.

We make this point regarding the GCM simulations in lines 147 to 159:

We note for clarity that these weathering values are all calculated with the same background CO_2 and global temperature and might therefore be better thought of as values of bulk "global weatherability," since Earth's climate would in reality adjust to bring the global weathering rate into balance with the background CO_2 outgassing rate if a change to continental geometry were to cause a large transient shift in weathering. In other words, these calculations are not intended to imply that the equilibrium global weathering rate has varied by this magnitude over Earth history (since the equilibrium global rate of weathering is set by outgassing), but instead to illustrate the degree to which changes in continental geometry can act as carbon cycle forcings under different assumptions about global weathering controls [Penman et al., 2020]. Several studies have identified changing "weatherability" as a potential major driver behind climate changes during the Phanerozoic Eon due to its influence on the equilibrium point of the carbon cycle and climate system [Kump and Arthur, 1997, Caves et al., 2016, Caves Rugenstein et al., 2019, Bergmann et al., 2025, Hayes et al., 2025].

———

However, it is true that we are using T as a CO_2 proxy. We discuss the fact that T is being used as a CO_2 proxy in lines 258 to 273:

Analyzing reconstructed CO_2 may seem like a more direct route for demonstrating a relationship between climate and paleogeography, since changes to silicate weathering fluxes should manifest as changes to CO_2 levels. While there do exist a number of CO_2 records over the Phanerozoic based on modeling and data compilations [Berner and Kothavala, 2001, Foster et al., 2017, Lenton et al., 2018], pre-Cenozoic CO_2 estimates are poorly constrained and often inconsistent Steinthorsdottir et al. [2025]. Given the large uncertainties associated with CO_2 paleo-records and the fact that Phanerozoic temperature variations are thought to mainly reflect CO_2 forcing [Royer et al., 2004, Mills et al., 2019], we choose to focus on GMST in this study. In any case, the reconstructed GMST of Judd et al. [2024] correlates strongly with (log-transformed) reconstructed CO_2 over most of the Phanerozoic [$r = 0.77$, $p < 0.01$, Judd et al., 2024], and our GCM ensemble displayed weak or non-existent relationships between global-mean surface temperature and both TLF and continental fragmentation (Fig. 4c, d). This increases our confidence that it is a reasonable first-order assumption that CO_2 and temperature were tightly linked over most of the Phanerozoic and that variations in continental geometry were unlikely to have a large direct (non-carbon-cycle-mediated) effect on surface temperatures, consistent with previous GCM ensemble studies [e.g., Otto-Bliesner, 1995, Lunt et al., 2016].

2. Two things from my-side of unclarity from the 1st round of comment.

1) What I mean 'terrestrial reverse weathering' is secondary mineral formation on land. This is normally called 'weathering congruency'. If congruency is low, a great share of dissolved phases would be precipitated as sec. min. before draining into the ocean, and apparently this would change the weathering flux.

Author response: Ah ha, this clarifies greatly, apologies for misunderstanding. Secondary mineral precipitation during the weathering process is very important. In fact, it is one of the main arguments for considering the possibility that weathering is runoff-

limited in some cases: The equilibrium point between dissolving silicate and precipitating secondary minerals sets the maximum concentration of weathering products in runoff, so if secondary mineral precipitation is occurring, the weathering is more likely to be runoff-limited. We note this in the introduction:

Lines 71 to 87 read:

One potential explanation for this discrepancy is that the feedback between climate and weathering may not be rooted directly in the kinetics of silicate dissolution, but is instead a result of the climate-dependence of the flux of water through the weathering zone [Maher and Chamberlain, 2014, Graham and Pierrehumbert, 2020]. Calculations of weathering rates that account for physicochemical interactions between water, dissolving silicates, and the secondary minerals (clays and silica) that precipitate during silicate weathering suggest that, depending on the local lithology, silicate weathering reactions can reach a chemical equilibrium where solutes in runoff are saturated, preventing further net silicate dissolution until unsaturated water flows through–a regime sometimes referred to as "chemostatic" or "runoff-limited" weathering [Maher et al., 2009, Maher and Chamberlain, 2014, Winnick and Maher, 2018, Hakim et al., 2021]. In this regime, climate influences weathering through its influence on hydrologic intensity, with greater fluxes of water leading to proportional increases in silicate weathering, largely independent of local temperature. Since global runoff has a much weaker dependence on temperature than the kinetic temperature dependence of silicate weathering, hydrologically regulated weathering leads to a weaker apparent temperature-dependence and a significantly less effective thermostat compared to silicate weathering based on laboratory measurements of silicate dissolution [Graham and Pierrehumbert, 2020].

——

We also carry out a set of calculations with a weathering model that explicitly accounts for secondary mineral precipitation, the Maher and Chamberlain [2014, MAC] model, with a plot of the results in the Appendix. This is basically a model of the transition between temperature-controlled weathering (when secondary mineral precipitation isn't occurring) and hydrology-controlled weathering (when secondary mineral precipitation is occurring).

Lines 446 to 457 describe this (also see Appendix section A.3):

The weathering parameterization we use in this study is based on the common formulation first suggested in Walker et al. [1981], sometimes referred to as the WHAK model [e.g., Kump and Volk, 1992, Graham and Pierrehumbert, 2020, Baum et al., 2022]. An alternative or extension to the WHAK model presented by Maher and Chamberlain [2014] (MAC) allows for mechanistic transitions between the weathering regimes explored here, with a temperature-controlled regime dominated by silicate dissolution kinetics and a hydrologically-regulated regime that occurs when secondary mineral precipitation in the weathering zone prevents solutes from accumulating to higher concentrations. Calculations with the MAC model display very similar patterns to results calculated with WHAK: parameter values with temperature-dependent weathering result in strong correlation between global weathering and TLF, while hydrology-dependent weathering results in a strong correlation with continental fragmentation (see Sec. A.3, Fig. A3).

2) What I mean about model validation is the choice of the silicate weathering equations and

parameters have been demonstrated with observed data/experimental data.

> **Author response:** The weathering equation we are using is essentially the standard representation of the process that is applied in most deep time models that incorporate silicate weathering into their carbon cycles. The parameter choices (i.e., temperature- vs. runoff-sensitivity) are poorly constrained, which is exactly the issue we are seeking to address in the paper (is weathering primarily dominated by temperature or runoff?). We have added a short note clarifying this:
>
> Lines 416 to 421 now read:
>
> This is a variant of the classic "WHAK" [Walker et al., 1981] formulation of continental silicate weathering, a widely used empirical expression to quantify CO_2 drawdown as a function of climate variables in geologic carbon cycle models [e.g., Pierrehumbert, 2010]. Models use a wide range of values for the temperature- and runoff-sensitivity parameters under investigation in this study, based on estimates from lab, field, geologic proxies, and theory [Walker et al., 1981, Berner et al., 1983, Sleep and Zahnle, 2001, Berner, 2006, Krissansen-Totton and Catling, 2017, Lenton et al., 2018, Godderis and Donnadieu, 2019].

3. From L372: I think the authors have introduced the silicate weathering equation nicely in the 'reply', but not in the 'Method' part of the manuscript. I would recommend authors briefly introduce to the audience the reasons for choosing this equation.

> **Author response:** The new lines we added in response to your point 2 seem to address this adequately.

References

Mark Baum, Minmin Fu, and Stephen Bourguet. Sensitive dependence of global climate to continental geometry. *Geophysical Research Letters*, page e2022GL098843, 2022.

Kristin D Bergmann, Francis A Macdonald, and Nicholas L Swanson-Hysell. The causes and consequences of ordovician cooling. *Annual Review of Earth and Planetary Sciences*, 53, 2025.

Robert A Berner. Geocarbsulf: a combined model for phanerozoic atmospheric o2 and co2. *Geochimica et Cosmochimica Acta*, 70(23):5653–5664, 2006.

Robert A Berner and Zavareth Kothavala. Geocarb iii: a revised model of atmospheric co 2 over phanerozoic time. *American Journal of Science*, 301(2):182–204, 2001.

Robert A. Berner, Antonio C. Lasaga, and Robert M. Garrels. The carbonate-silicate geochemical cycle and its effect on atmospheric carbon dioxide over the past 100 million years. *American Journal of Science*, 283:641–683, 1983.

Jeremy K Caves, Adam B Jost, Kimberly V Lau, and Kate Maher. Cenozoic carbon cycle imbalances and a variable weathering feedback. *Earth and Planetary Science Letters*, 450:152–163, 2016.

Jeremy K Caves Rugenstein, Daniel E Ibarra, and Friedhelm von Blanckenburg. Neogene cooling driven by land surface reactivity rather than increased weathering fluxes. *Nature*, 571(7763): 99–102, 2019.

Y. Donnadieu, Y. Godderis, G. Ramstein, A. Nedelec, and J. Meert. A 'Snowball Earth' climate triggered by continental break-up through changes in runoff. *Nature*, 428(6980):303–306, mar 18 2004. ISSN 0028-0836.

Y Donnadieu, Y Goddéris, R Pierrehumbert, Gilles Dromart, Frédéric Fluteau, and R Jacob. A geoclim simulation of climatic and biogeochemical consequences of pangea breakup. *Geochemistry, Geophysics, Geosystems*, 7(11), 2006a.

Y Donnadieu, R Pierrehumbert, R Jacob, and Frédéric Fluteau. Modelling the primary control of paleogeography on cretaceous climate. *Earth and Planetary Science Letters*, 248(1-2):426–437, 2006b.

Gavin L Foster, Dana L Royer, and Daniel J Lunt. Future climate forcing potentially without precedent in the last 420 million years. *Nature communications*, 8(1):1–8, 2017.

Yves Godderis and Yannick Donnadieu. A sink-or a source-driven carbon cycle at the geological timescale? relative importance of palaeogeography versus solid earth degassing rate in the phanerozoic climatic evolution. *Geological Magazine*, 156(2):355–365, 2019.

Yves Godderis, Yannick Donnadieu, Celine Dessert, Bernard Dupre, Frederic Fluteau, Louis M Francois, Joseph Meert, Anne Nedelec, and Gilles Ramstein. Coupled modeling of global carbon cycle and climate in the neoproterozoic: links between rodinia breakup and major glaciations. *Cr Geosci*, 339(3-4):212–222, 2007. doi:10.1016/j.crte.2005.12.002.

Yves Goddéris, Yannick Donnadieu, Guillaume Le Hir, Vincent Lefebvre, and Elise Nardin. The role of palaeogeography in the phanerozoic history of atmospheric co2 and climate. *Earth-Science Reviews*, 128:122–138, 2014.

RJ Graham and RT Pierrehumbert. Thermodynamic and energetic limits on continental silicate weathering strongly impact the climate and habitability of wet, rocky worlds. *Astrophysical Journal*, 896(2), 2020.

Khushboo Gurung, Katie J Field, Sarah A Batterman, Simon W Poulton, and Benjamin JW Mills. Geographic range of plants drives long-term climate change. *Nature Communications*, 15(1):1805, 2024.

Kaustubh Hakim, Dan J Bower, Meng Tian, Russell Deitrick, Pierre Auclair-Desrotour, Daniel Kitzmann, Caroline Dorn, Klaus Mezger, and Kevin Heng. Lithologic controls on silicate weathering regimes of temperate planets. *The Planetary Science Journal*, 2(2):49, 2021.

Nick R Hayes, Daniel J Lunt, Yves Goddéris, Richard D Pancost, and Heather Buss. Modelling the impact of palaeogeographical changes on weathering and co2 during the cretaceous–eocene period. *Climate of the Past*, 21(12):2601–2618, 2025.

Emily J Judd, Jessica E Tierney, Daniel J Lunt, Isabel P Montañez, Brian T Huber, Scott L Wing, and Paul J Valdes. A 485-million-year history of earth's surface temperature. *science*, 385(6715): eadk3705, 2024.

Preston Cosslett Kemeny, Alex Krause, and Lee Kump. A historical perspective on long-term global biogeochemical box models. *Hydrothermal Circulation and Seawater Chemistry: Links and Feedbacks*, pages 263–295, 2025.

Joshua Krissansen-Totton and David C Catling. Constraining climate sensitivity and continental versus seafloor weathering using an inverse geological carbon cycle model. *Nature communications*, 8(1):1–15, 2017.

Lee Kump and Tyler Volk. Gaia's garden and BLAG's greenhouse: global biogeochemical climate regulation. In *Scientists on Gaia*, pages 191–199. MIT Press, 1992. ISBN 978-0-262-19310-8.

Lee R Kump and Michael A Arthur. Global chemical erosion during the cenozoic: Weatherability balances the budgets. In *Tectonic uplift and climate change*, pages 399–426. Springer, 1997.

Timothy M Lenton, Stuart J Daines, and Benjamin JW Mills. Copse reloaded: an improved model of biogeochemical cycling over phanerozoic time. *Earth-Science Reviews*, 178:1–28, 2018.

Jack Longman, Benjamin JW Mills, and Andrew S Merdith. Limited long-term cooling effects of pangaean flood basalt weathering. *Nature Communications*, 16(1):4813, 2025.

Daniel J Lunt, Alex Farnsworth, Claire Loptson, Gavin L Foster, Paul Markwick, Charlotte L O'Brien, Richard D Pancost, Stuart A Robinson, and Neil Wrobel. Palaeogeographic controls on climate and proxy interpretation. *Climate of the Past*, 12(5):1181–1198, 2016.

K Maher and CP Chamberlain. Hydrologic regulation of chemical weathering and the geologic carbon cycle. *Science*, 343(6178):1502–1504, 2014.

Kate Maher, Carl I Steefel, Art F White, and Dave A Stonestrom. The role of reaction affinity and secondary minerals in regulating chemical weathering rates at the santa cruz soil chronosequence, california. *Geochimica et Cosmochimica Acta*, 73(10):2804–2831, 2009.

Benjamin JW Mills, Alexander J Krause, Christopher R Scotese, Daniel J Hill, Graham A Shields, and Timothy M Lenton. Modelling the long-term carbon cycle, atmospheric co2, and earth surface temperature from late neoproterozoic to present day. *Gondwana Research*, 67:172–186, 2019.

Benjamin JW Mills, Yannick Donnadieu, and Yves Goddéris. Spatial continuous integration of phanerozoic global biogeochemistry and climate. *Gondwana Research*, 2021.

Elise Nardin, Yves Goddéris, Yannick Donnadieu, Guillaume Le Hir, Ronald C Blakey, Emmanuelle Pucéat, and Markus Aretz. Modeling the early paleozoic long-term climatic trend. *Bulletin*, 123 (5-6):1181–1192, 2011.

Bette L Otto-Bliesner. Continental drift, runoff, and weathering feedbacks: Implications from climate model experiments. *Journal of Geophysical Research: Atmospheres*, 100(D6):11537–11548, 1995.

Yuem Park, Pierre Maffre, Yves Goddéris, Francis A Macdonald, Eliel SC Anttila, and Nicholas L Swanson-Hysell. Emergence of the southeast asian islands as a driver for neogene cooling. *Proceedings of the National Academy of Sciences*, 117(41):25319–25326, 2020.

Donald E Penman, Jeremy K Caves Rugenstein, Daniel E Ibarra, and Matthew J Winnick. Silicate weathering as a feedback and forcing in earth's climate and carbon cycle. *Earth-Science Reviews*, page 103298, 2020.

Raymond T. Pierrehumbert. *Principles of Planetary Climate*. Cambridge University Press, 2010.

Dana L Royer, Robert A Berner, Isabel P Montañez, Neil J Tabor, David J Beerling, et al. Co˜ 2 as a primary driver of phanerozoic climate. *GSA today*, 14(3):4–10, 2004.

Norman H Sleep and Kevin Zahnle. Carbon dioxide cycling and implications for climate on ancient earth. *Journal of Geophysical Research: Planets*, 106(E1):1373–1399, 2001.

Margret Steinthorsdottir, Isabel P Montañez, Dana L Royer, Benjamin JW Mills, and Bärbel Hönisch. Phanerozoic atmospheric co2 reconstructed with proxies and models: Current understanding and future directions. *Treatise on Geochemistry*, pages 467–492, 2025.

J. C. G. Walker, P. B. Hays, and J. F. Kasting. A negative feedback mechanism for the long term stabilization of Earth's surface temperature. *Journal of Geophysical Research*, 86(NC10): 9776–9782, 1981.

Matthew J Winnick and Kate Maher. Relationships between co2, thermodynamic limits on silicate weathering, and the strength of the silicate weathering feedback. *Earth and Planetary Science Letters*, 485:111–120, 2018.

Thomas R Worsley and David L Kidder. First-order coupling of paleogeography and co2, with global surface temperature and its latitudinal contrast. *Geology*, 19(12):1161–1164, 1991.

Thomas R Worsley, Thomas L Moore, Carmen M Fraticelli, and Christopher R Scotese. Phanerozoic co2 levels and global temperatures inferred from changing paleogeography. 1994.

Feifei Zhang, Guang-Yi Wei, Pierre Maffre, Ziheng Li, Jianlin Zhou, Alexandre Pohl, Yi-Bo Lin, Maya Elrick, Keyi Cheng, Philip AE Pogge von Strandmann, et al. Lithium isotope evidence for enhanced continental weathering triggering the late paleozoic greenhouse-to-icehouse climate transition. *National Science Review*, 13(8):nwag168, 2026.

Reviewer #1 (Remarks to the Author)

I thank the authors for adressing the comments raised by myself and other reviewers. Please find my thoughts on each comment below in green. I am generally content with the changes the authors have made but, in some instances, the original comments could/should be addressed with more consideration. I particularly like the new Figure 4 and the new discussion on the sensitivity of the result to choice of paleogeographic reconstruction–both improved the manuscript.

This remains an incredibly interesting study, using an original approach to study what physical processes contributed to the large-scale evolution of Earth's climate across time. I highly recommend publication; resubmit with minor corrections.

Regards,
Pam Vervoort

1.1 General Comments

The first part demonstrates clearly that either fragmentation or TLF is important, depending on the dominant weathering rates control. These results are interesting, and it would benefit from a more extended investigation into why this is. On Lines 138–150 reasons are given for why temperature-dependent weathering is mostly linked to TLF while runoff-dependent weathering is mostly linked to fragmentation. While straightforward, the reader needs to see the evidence to back this up. In current form, the reader cannot judge how temperature and runoff change with fragmentation and TLF. For example, I imagine that temperature and fragmentation are not entirely decoupled as fragmentation affects ocean-atmosphere interaction. Especially the statement "[. . .] changing TLF does not tend to lead to large changes in runoff, [. . .]" needs further explanation; this is not per se intuitive. An increase in TLF can also increase runoff as more land is located in high-precipitation regions.

Author response: We have added a new figure (figure 4) showing how runoff, global mean surface temperature, and global-mean land temperature all vary with TLF and continental fragmentation. We altered lines 152 to 171 to read: …

Reviewer response: New Fig 4 is a nice addition that clarifies the conclusions drawn. It is clear why there is a relation between TLF and land surface temp, however, I'm still unsure why TLF does not influence runoff. I can see R^2=0 in panel a but I'm looking for a physical explanation as opposed to simply showing 'no correlation'. Would an increase in TLF not lead to more runoff with more land in high-precipitation regions? I'm actually not sure about the answer but substantiating this model behaviour with 'real world' or modelled physical mechanisms would help me and other readers understand. This point is important because the result of the

papers rely on this. (For context: I'm thinking about the Amazon River. Amazon land lies in the tropical region and it's one of the rivers with the largest runoff rates today).

1. The authors assume a direct link between CO_2 and temperature which is perhaps somewhat simplistic. While it is true for most of the Phanerozoic, Judd et al. (2024) also point out that there is no strong correlation between CO_2 and GMST during the Mesozoic (250–66 Ma). Under the same CO_2, GMST can differ when the land mass configuration is different. Lands have a different heat capacity and albedo profile than oceans, and changes in heat distribution by ocean currents change with landmass. In addition, some paleogeographies are more prone to develop ice sheets with can lead to further decoupling of GMST and CO_2. This point needs to be addressed in my opinion to back up why GMST can be used to infer weathering regimes. A major benefit of the idealized GCM ensemble is that you could identify the extent to which landmass distribution affects the global temperature (under fixed CO_2) and use that to discuss the implications for the GMST–CO_2 relationship.

Author response: In addition to the addition of Figure 4 and discussion of the relationships between temperature, TLF, and fragmentation noted above, we have now added the following to lines 235 to 242: …

Reviewer response: Thanks for clarifying. I appreciate acknowledgement of the uncertainties surrounding the GMST-CO_2 relation. Stating the positive correlation between GMST and CO_2 is a first-order assumption is necessary. The new Fig 4cd suggest that paleogeography has minimal influence on the GMST though panel c shows that there is still a positive R^2=16% relation between TLF and GMST. Is this statistically significant? I am still of the opinion that a more in-depth discussion or analysis of how paleogeography, especially latitudinal landmass distribution, affects GMST (all else being equal) is appropriate. E.g. discuss existing and very relevant literature (e.g. Worsley et al., 1994 or Lunt et al., 2016, and others)?

Further, I am wondering about the role of background climate state. The Phanerozoic has seen large climate change. Paleoclimate proxy records suggest that at higher global temperatures, the latitudinal temperature gradient is less steep and hydrological cycling is more intense, presumably with consequences for the importance of TLF and fragmentation in controlling the weathering strengths?

Author response: Latitudinal temperature gradient has varied by a bit less than a factor of two over the past 95 million years, which encompasses the hottest period of the Phanerozoic, corresponding to a minimum equator-to-pole temperature gradient of 15.6 C (Gaskell et al., 2022). If we assume a fairly weak temperature feedback with an e-folding temperature of 30 C (corresponding to $k = 1/30\ C^{-1}$), then teleporting a continent from pole to equator would multiply weathering by a factor of $\exp 15.6/30 \approx 1.7$. With a stronger e-folding temperature of 15 C ($k=1/15\ C^{-1}$),

movement from pole to equator would increase weathering by a factor exp 15.6/15 ≈ 5.9. In other words, with the weakest expected equator-to-pole temperature gradient, TLF should still significantly impact weathering rates even if the temperature feedback is weak, and if the temperature feedback is strong, the effect can still be quite large.

Regarding changes to hydrological cycling, if weathering is primarily temperature-dependent, then more intense hydrological cycling won't have much impact on weathering. If weathering is primarily hydrologically-mediated, then more intense hydrological cycling will lead to increased weathering, since that is the basic feedback mechanism. Unless the background temperature state is somehow influencing the feedback mechanism, it shouldn't affect the point that fragmentation is important for hydrologically-controlled weathering but not for temperature-controlled weathering. In a set of calculations not included in the manuscript, we looked into this a bit using a subset of the idealized paleogeographies in the paper (just 10 instead of 40), evaluating the correlations at 500 ppm and 2000 ppm (vs. 1000 ppm in the paper); the same bulk correlations appear.

Reviewer response: Indeed, I was mainly wondering if the background climate state influences the feedback mechanism because a different meridional temperature gradient affects hydrology and temperature patterns. It's important information that the results at 500 ppm and 2000 ppm are comparable to those you find at 1000 ppm! In fact, that would be useful to mention in the manuscript.

2. The authors assume a direct link between (high) weathering and (low) CO_2. While intuitive, it may not always be the case. For instance, a recent paper (Hülse and Ridgwell, 2025) shows that CO_2 drawdown from silicate weathering may be supplemented by enhanced CO_2 drawdown from concurrent organic carbon burial, i.e. carbon burial is also important in controlling CO_2, especially during times of low oxygenation, and may destabilize the thermostat. Further, some geological intervals are characterized by high CO_2 as well as high weathering rates that can be sustained for millions of years because CO_2 emissions (e.g. volcanism) exceed the weathering rate. Whether these processes are relevant for Phanerozoic intervals on the long-timescales studies here should be discussed, substantiated, or refuted.

Author response: This point, while well-taken, is a fundamental question deserving of its own study. To briefly address why we think the assumption of equality between degassing and silicate weathering is reasonable for our study: The Hülse and Ridgwell paper only carries simulations out to 500,000 years, and based on Fig. 3 in their paper it appears that regardless of the level of transient cooling from the carbon cycle instability, the CO_2 values are converging back to the equilibrium level that existed before the imposed climate perturbation. It doesn't appear that the instability suggested in the paper actually impacts the long-term equilibrium state of the

atmosphere unless the climate hits a bifurcation point and globally glaciates during the transient cooling, and the transient cooling is maximized at lower oxygen values than we have seen over most of the Phanerozoic. Re: the point about CO2 emissions exceeding weathering for millions of years, this is theoretically possible but it would lead to very large excursions in climate that would likely eliminate complex life, see e.g., Berner and Caldeira, 1997; Broecker and Sanyal, 1998; or Zeebe and Caldeira, 2008. That said, we have added a brief mention of these issues. Lines 52 to 59 now read:

Although transient imbalances between CO2 inputs and outputs due to organic carbon interactions [Hülse and Ridgwell, 2025] or limitations in silicate mineral supply [Kump, 2018] may have played important roles in Earth's climate evolution, weathering-based coupling between atmospheric CO2 removal and background climate appears to play a key role in maintaining the quasi-equilibrium in the carbon cycle necessary to avoid frequent excursions to extremely high or low surface temperatures that would have threatened the existence of complex life on Earth during the Phanerozoic [Broecker and Sanyal, 1998, Zeebe and Caldeira, 2008]

Reviewer response: Thanks for clarifying, happy with the changes.

All in all, the manuscript is incredibly interesting and could serve as a significant scientific contribution. I would very much like to see this work published, but in the current iteration, the uncertainties are not yet sufficiently addressed and are perhaps too large to justify the conclusion that the weathering regime changed throughout the Phanerozoic based solely on the correlation between landmass distribution and GMST. Nonetheless, I think there is potential for improvement. For instance, using the idealized GCM ensemble to quantify the relation between GMST and landmass. I would also like to highlight that a full series of Phanerozoic GCM (HadCM3) simulations exists (Valdes et al., 2020) with published climatological fields of runoff and temperate that may be interesting to consider in this study.

Author response: We appreciate the positive and constructive comments. We quantified the relationship between GMST and landmass using the GCM results like you suggested. We elected not to analyze the Valdes dataset in this study; however it is a point we have noted for follow-up, and we appreciate it.

Reviewer response: Thanks, happy with the changes.

New minor comments:

L. 100: "*though this relies on assumptions like weakly varying outgassing*." And the assumed simplified relation between CO2 and global mean temperature? And, especially further back in time, uncertainties in paleogeographic reconstructions?

L. 172: "*These distinctive differences in the response of the ~~carbon cycle~~ to continental geometry naturally lead to a hypothesis*:" Do you mean '…response of the weathering feedback to continental geometry….'?

References

Worsley, T.R. and Kidder, D.L., 1991. First-order coupling of paleogeography and CO2, with global surface temperature and its latitudinal contrast. Geology, 19(12), pp.1161-1164.

Lunt, D.J., Farnsworth, A., Loptson, C., Foster, G.L., Markwick, P., O'Brien, C.L., Pancost, R.D., Robinson, S.A. and Wrobel, N., 2016. Palaeogeographic controls on climate and proxy interpretation. *Climate of the Past*, *12*(5), pp.1181-1198.
